# Bioinspired NiCoO_2_ nanocomposites derived from *Moringa oleifera* as a recyclable catalyst for the synthesis of pyrazolopyrano[2,3-d]pyrimidine with potent antimicrobial efficiency

**DOI:** 10.1186/s11671-026-04800-6

**Published:** 2026-08-03

**Authors:** Prakash Kokare, Avdhut Kadam, Ashwini More, Vijay Shinde, Vishvanath Ghanwat, Nilesh Pandit, Chaitali Bagade, Avinash Survase, Komal Mali, Santosh Kamble

**Affiliations:** 1 Department of Chemistry, Yashavantrao Chavan Institute of Science, Constituent College of Karmaveer Bhaurao Patil University, Satara, 415001 Maharashtra India; 2 Department of Chemistry, Shivaji University, Kolhapur, 416004 Maharashtra India; 3 Department of Microbiology, Yashavantrao Chavan Institute of Science, Constituent College of Karmaveer Bhaurao Patil University, Satara, 415001 Maharashtra India; 4 Department of Chemistry, Sadguru Gadage Maharaj College, Karad, 415124 Maharashtra India; 5 Karmaveer Bhaurao Patil University, Satara, 415001 Maharashtra India; 6 Sambhajirao Kadam College, Deur, 415524 Maharashtra India; 7 Department of Physics, Yashavantrao Chavan Institute of Science, Constituent College of Karmaveer Bhaurao Patil University, Satara, 415001 Maharashtra India

**Keywords:** NiCoO_2_ nanocomposite, *Moringa oleifera* leaves extract, Antimicrobial activity, Ultrasonication, Heterogeneous catalysis, Pyrazolopyrano[2,3-d]pyrimidine

## Abstract

A green synthetic approach was developed for the preparation of a NiCoO₂ nanocomposite using an aqueous extract of *Moringa oleifera* as both a reducing and stabilizing agent. The synthesized NiCoO₂ nanocomposite exhibited high crystallinity and measurable antimicrobial activity. Detailed characterization using XRD, UV–visible spectroscopy, fluorescence spectroscopy, FTIR, BET, SEM, EDS, elemental mapping, and TEM analyses confirmed its crystalline structure, elemental composition, thermal stability, surface functional groups, and morphological features. The nanocomposite and the synthesized products were evaluated for antimicrobial activity against *S. aureus*, *B. cereus*, *P. vulgaris*, and *S. typhimurium*, demonstrating inhibitory effects against the tested microorganisms. The NiCoO₂ nanocomposite was also employed as a heterogeneous catalyst in a one-pot multicomponent condensation reaction of aldehydes, hydrazine hydrate, barbituric acid, and ethyl acetoacetate under ultrasonic conditions, affording pyrazolopyrano[2,3-d]pyrimidine derivatives in yields of up to 98% within short reaction times. The catalyst retained its activity over five consecutive cycles, indicating good recyclability. Furthermore, the synthesized heterocycles exhibited antimicrobial activity against both Gram-positive and Gram-negative bacterial strains. Molecular docking studies revealed that all synthesized compounds (1–11) exhibited favorable binding affinities toward the 4COX enzyme, with docking scores higher than that of the reference drug indomethacin, suggesting their potential for further biological evaluation.

**Graphical abstract:**

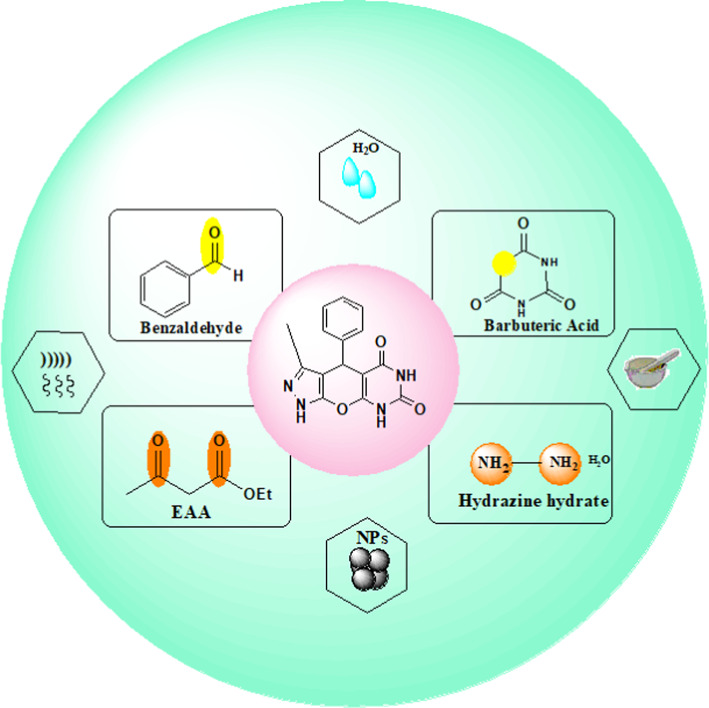

**Supplementary Information:**

The online version contains supplementary material available at 10.1186/s11671-026-04800-6.

## Introduction

Modern chemical research is progressively shifting toward advanced methodologies that prioritize the principles of green chemistry, focusing on environmentally benign, energy-efficient, and sustainable reaction protocols [1]. Nowadays, nanocatalysts are a growing field, significant in nearly all kinds of catalytic organic transformations, and constitute an essential tool in green chemistry, as they aid in reducing the use of hazardous chemical processes and open up synthetic pathways to desired products while adopting a sustainable approach [2–4] Nanocatalysts are present in different forms, such as metal nanoparticles [5], metal-oxide nanoparticles, supported nanoparticles [6], nanocomposites, and bimetallic nanoparticles [7, 8]. With the rapid growth of nanotechnology, researchers now have precise control over the composition and design of these catalytic systems. In particular, customized nanocomposites represent highly promising heterogeneous catalysts for organic transformations [9, 10].

Nanocomposites are a combination of two different metallic elements that demonstrate unique catalytic properties due to synergistic electronic and geometric effects, often surpassing their monometallic counterparts in activity, selectivity, and cost efficiency [11]. Their catalytic performance can be finely tuned through variation in composition, particle size, and morphology, including alloy, core–shell, and hollow designs. However, despite significant progress in the synthesis of metal oxide nanomaterials, many existing protocols still rely on toxic chemicals, harsh reaction conditions, and non-renewable resources, limiting their environmental compatibility and scalability [12, 13].

To address these challenges, greener and more sustainable approaches for nanocomposite synthesis are increasingly being explored. Nickel and cobalt were selected for the nanocomposite due to their excellent redox activity, synergistic catalytic properties, high surface area in nanostructured forms, and cost-effectiveness compared to other transition metals. Nickel cobalt oxide (NiCoO₂) nanocomposites were used as catalysts in an environmentally benign, aqueous-phase four-component synthesis of pyrazolopyrano[2,3-d]pyrimidine derivatives. These nanocomposites can be synthesized through a range of methods, such as co-reduction and sequential reduction, and may be employed either unsupported or immobilized on substrates, including clays, zeolites, fibres, polymers, and graphene.

Among sustainable synthesis routes, plant-mediated approaches have gained considerable attention because they provide eco-friendly reducing and stabilizing agents derived from renewable resources. Mahardika Yoga Darmawan et al. reported a green synthesis of an Fe₃O₄/Ag nanocomposite using *Moringa oleifera* [14]. In the present work, we synthesized a NiCoO₂ nanocomposite using *Moringa oleifera* leaf extract. This leaf extract functions effectively as a green reducing and stabilizing agent in the eco-friendly synthesis of the NiCoO₂ nanocomposite. It is rich in phytochemicals, which exhibit properties such as reduction and capping, along with biological activity [15–17]. Its leaves are traditionally used in ethnomedicine due to their demonstrated antimicrobial, anti-inflammatory, and antioxidant activities [18–20]. Moreover, nanocomposites of Ni and Co nanoparticles supported on inorganic oxides have demonstrated their effectiveness in selective hydrogenation processes, underscoring the versatility of these 3d transition metal-based heterogeneous catalysts in diverse organic reactions [21].

In parallel with catalyst development, multicomponent reactions (MCRs) have emerged as an efficient and sustainable strategy in modern organic synthesis. Multicomponent reactions are powerful one-pot processes in which three or more reactants combine to form a single product [22]. Compared to conventional stepwise syntheses, MCRs offer several advantages, including higher overall yields, simplified work-ups, reduced waste, improved atom economy, fewer synthetic steps, and lower solvent consumption, aligning well with the principles of green chemistry [23–26]. Four-component reactions are particularly important in synthetic chemistry, as they involve the straightforward mixing of four reactants and provide high efficiency, high atom economy, and high energy efficiency [27]. These reactions have emerged as an important tool in green chemistry, offering sustainable and eco-friendly applications compared to traditional organic reactions due to their lower energy consumption and reduced generation of waste and hazardous chemicals [28–30].

To further improve the efficiency of these reactions, ultrasound sonication was employed as a green activation technique. Ultrasonic waves promote efficient mixing, rapid mass transfer, and cavitation effects, which accelerate the reaction rate and improve product yield. This technique also supports green reaction conditions by reducing reaction time, minimizing solvent usage, and eliminating the need for conventional heating.

There is growing interest in developing environmentally friendly methods to synthesize important molecules such as heterocyclic compounds. Heterocyclic compounds are essential to modern science, from the interior workings of life to the design of life-saving drugs, eco-friendly agrochemicals, and advanced materials [31–33]. Their structural diversity, natural compatibility, and synthetic convenience make them central to innovation across chemistry, biology, and technology.

Among these, pyrazolopyrano[2,3-d]pyrimidine derivatives are especially significant because of their diverse biological activities. Therefore, nanocomposite-based catalysis offers an efficient and sustainable approach for the synthesis of these heterocyclic compounds. Herein, we employed a NiCoO₂ nanocomposite for the synthesis of pyrazolopyrano[2,3-d]pyrimidine derivatives using aromatic aldehydes, ethyl acetoacetate, hydrazine hydrate, and barbituric acid in an ethanol–water (1:1) solvent system under ultrasonication with excellent yield [34]. These synthesized pyrazolopyrano[2,3-d]pyrimidines exhibit various biological activities, including anticancer, antibacterial, antimicrobial, antifungal, and anti-inflammatory activities.

In the current work, we performed a multicomponent reaction for the synthesis of the desired product. Existing literature lacks studies on the integration of such nanocomposites as reusable catalysts in the synthesis of biologically active pyrazolopyrano[2,3-d]pyrimidine derivatives under ultrasonication. Therefore, this study aims to (i) develop a sustainable method for synthesizing a NiCoO₂ nanocomposite using *Moringa oleifera* plant extract, (ii) characterize the nanocomposite using advanced techniques, (iii) evaluate its catalytic potential in the synthesis of pyrazolopyrano[2,3-d]pyrimidines under ambient conditions, and (iv) examine the antimicrobial properties of the synthesized derivatives.

## Materials and methods

### Materials

*Moringa oleifera* plant leaves were collected from Satara, Maharashtra, India. Potassium hydroxide (KOH, 1 M, purity 99%), nickel chloride (NiCl₂, 1 M, purity 99%), and cobalt nitrate [Co(NO₃)₂, 1 M, purity 99%], aldehydes (1 mmol, purity 99%), hydrazine hydrate (1 mmol, purity 99%), ethyl acetoacetate (1 mmol, purity 99%), and barbituric acid (1 mmol, purity 99%) were of AR grade and purchased from Sigma-Aldrich (St. Louis, MO) and used as received without further purification in distilled water.

### Instrumentation

The synthesized nanocomposites were characterized using the X-ray diffraction (XRD) technique with a BRUKER AXS D8 Advanced XRD using Cu radiation (Cu K_α_λ = 1.54 A^°^). The optical investigation of the nanocomposites was carried out using UV–visible spectroscopy. The optical properties of the nanocomposites were studied using fluorescence spectroscopy, which was carried out at ICT, Mumbai. Fourier transform infrared spectroscopy (Lambda Scientific FTIR-7600 spectrometer) was used for the functional analysis of the molecules. The textural properties of the nanocomposites were analyzed using BET analysis, which was performed at Jain University, Jakkasandra Ramanagaram, Bangalore. The morphology of the nanocomposite was analyzed using a scanning electron microscope (SEM, JEOL JSM-IT-200) at Y.C.I.S., Satara. The shape and morphology of the synthesized nanocomposites were examined using Transmission Electron Microscopy (TEM), with analysis carried out at Shivaji University, Kolhapur. The ^1^H NMR and ^13^C NMR spectra were recorded on a Bruker Avance 400 MHz & 101 MHz, respectively, spectrometer using chloroform (DMSO) as the solvent at Solapur University. Mass spectrometric analysis was conducted at the Institute of Chemical Technology (ICT), Mumbai.

## Experimental

### Preparation of plant extract

The young and mature leaves of *Moringa oleifera* were thoroughly washed with distilled water, and the excess water was removed by oven-drying at 100 °C for 1 h. The dried leaves were then finely powdered and subjected to extraction using a Soxhlet apparatus with water as the solvent for 2.5 h.

### Preparation of NiCoO_2_ nanocomposite

A 100 mL round-bottom flask was charged with 10 mL of 1.0 M NiCl₂ solution and 10 mL of 1.0 M Co(NO₃)₂ solution. To this mixed-metal precursor solution, a 25 mL aliquot of *Moringa oleifera* plant extract was added dropwise under continuous ultrasonic irradiation to facilitate homogeneous nucleation and effective phytochemical-mediated reduction/stabilization. Subsequently, 25 mL of 1.0 M KOH solution was introduced gradually until complete precipitation was achieved at pH 10.5, thereby providing a sufficiently alkaline medium for metal hydroxide formation. The resulting reaction mixture was further subjected to ultrasonic treatment for 2 hrs using an ultrasonicator operating at a frequency of 40 kHz and a power output of 100 W, ensuring enhanced dispersion and controlled particle growth. Thereafter, the precipitate was isolated by centrifugation and washed three times with distilled water to remove residual impurities and unreacted species. The recovered nanocomposite product was then oven-dried and subsequently calcined at 400 °C for 6 h to improve crystallinity and phase stability. The concentrations of the two metal precursors were systematically optimized through preliminary experimental trials to achieve maximum catalytic efficiency, minimal by-product formation, and stable nanocomposite generation, in accordance with previously reported procedures [35].

### Antimicrobial activity

Bacterial inocula were prepared in sterile saline water. Nutrient agar plates served as the growth medium for the bacterial strains. Cultures of *S. aureus*, *B. cereus*, *P. vulgaris*, and *S. typhimurium* were uniformly overlaid on nutrient agar plates. Wells of 6 mm diameter were generated using a cork borer. A 100 µg/mL concentration of the synthesized material was suspended in sterile distilled water and introduced into the wells using a micropipette. The spread petri plates were incubated at 37 °C for 24 h to assess antibacterial activity [36, 37].

### Synthesis pyrazolopyrano[2,3-d]pyrimidines

In a 50 mL round-bottom flask, equimolar quantities of an aromatic aldehyde (1 mmol), ethyl acetoacetate (1 mmol), hydrazine hydrate (1 mmol), and barbituric acid (1 mmol) were combined in the presence of the NiCoO₂ nanocomposite catalyst (5 mol%). The reaction mixture was dissolved in 10 mL of a 1:1 (v/v) water–ethanol solvent system and subjected to ultrasonication at 40 °C. The progress of the reaction was monitored by thin-layer chromatography (TLC) using n-hexane: ethyl acetate (8:2, v/v) as the mobile phase. Upon completion of the reaction, the resulting product was isolated by filtration using Whatman No. 41 filter paper. The crude product was subsequently recrystallized using ethanol to obtain the purified compound, and its purity was further confirmed by TLC analysis. The recrystallized and purified product was then subjected to structural characterization using FT-IR, ^1^H NMR, and ^13^C NMR spectroscopy.

### Molecular docking studies

**Fig. 1:**
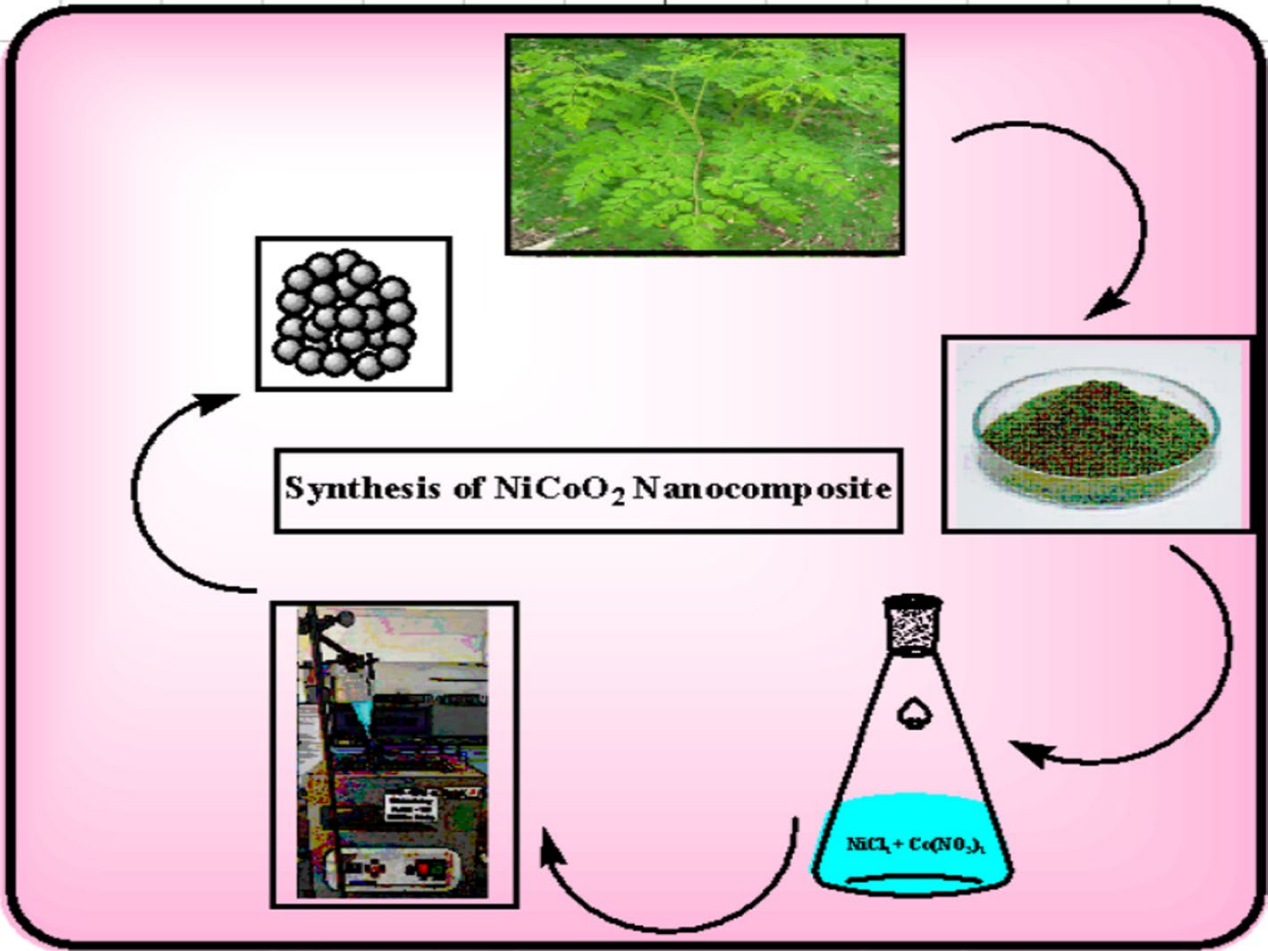
Schematic for the synthesis of NiCoO_2_ nanocomposite

The drug development and design process is becoming increasingly complex because of the high costs and lengthy duration associated with it. Computational methods have therefore developed rapidly in recent years, as they offer many benefits, such as reduced time, cost, and resource use compared with experimental approaches. One of the techniques used to predict the shape and orientation of a ligand at a particular region of a protein target is called molecular docking. Docking research aims to generate comprehensive three-dimensional models of the structures of the studied compounds to provide insights into their biological activity [38]. We retrieved Cyclooxygenase-2 (PDB ID: 4COX) from the RCSB Protein Data Bank [39] and then processed and cleaned the protein structure. The 4COX protein consists of two polypeptide chains (A and B). Among these, chain A was chosen as the macromolecule for preparation purposes. The chemical structures of compounds (1–12) were drawn using ChemDraw Professional 16.0, and AutoDock Vina 4.2 implemented in PyRx 0.8 was used to perform the molecular docking studies [40]. Molecular docking studies of the compounds synthesized in this study and indomethacin were carried out against COX-2. The size of the grid box was determined based on the native inhibitor, and all compounds (1–12) were evaluated using the same docking parameters during the molecular docking studies. Images of the protein–ligand complexes generated from the molecular docking studies were visualized using BIOVIA Discovery Studio Visualizer [41] (Fig. 1).

## Result and discussion

### Mechanism for the synthesis of NiCoO_2_ nanocomposite

The green synthesis of NiCoO_2_ nanocomposites using *Moringa oleifera* leaf extract is driven by its bioactive phytochemicals, including flavonoids, phenolics, terpenoids, tannins, and proteins. Flavonoids and phenolics function as reducing agents, converting Ni^2+^ and Co^2+^ ions into nanoparticles, while terpenoids and proteins act as capping and stabilizing agents, preventing aggregation and controlling particle size. Functional groups such as –OH and –COOH also chelate metal ions, facilitating uniform nucleation and growth. This combined mechanism enables the eco-friendly synthesis of Ni/Co nanocomposites with controlled morphology and enhanced stability [42].

### Characterization of NiCoO_2_ nanocomposite

#### XRD

**Fig. 2:**
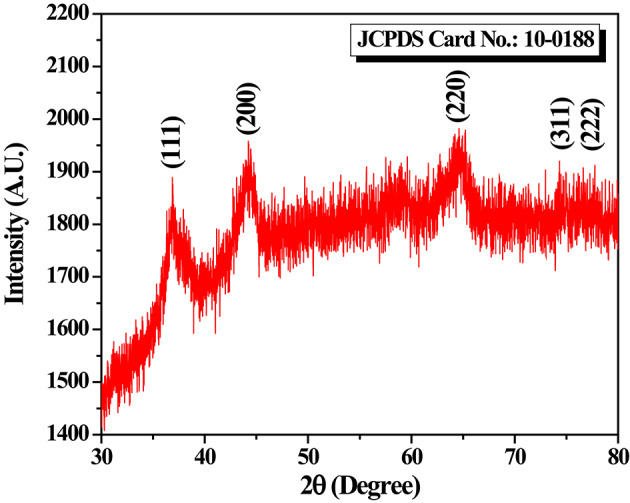
XRD pattern of NiCoO_2_nanocomposite

The structural and crystallographic nature of the synthesized NiCoO₂ nanocomposites was analyzed using XRD, as shown in Fig. 2. The pattern confirms the highly crystalline nature of the sample, indicating its phase purity and nanocrystallinity. The peaks in the XRD pattern visible at 2θ values of 36.8°, 42.82°, 61.79°, 73.99°, and 77.99° correspond to the (hkl) planes of (111), (200), (220), (311), and (222), respectively, for NiCoO_2_ nanocomposites. The observed peaks are aligned with those of the standard NiCoO_2_ (JCPDS card no. 10–0188). The crystallite size of the NiCoO_2_ nanocomposite was estimated from XRD using the Debye–Scherrer equation and was found to be 30 nm. The Debye–Scherrer formula is used to estimate the crystallite size from XRD peaks, and the formula is D = Kλ/βcosθ, where D, K, λ, β, and θ are the average crystallite size, Scherrer constant (0.9), wavelength of the X-ray, full width at half maximum, and Bragg’s diffraction angle, respectively [43].

#### UV visible spectroscopy

**Fig. 3:**
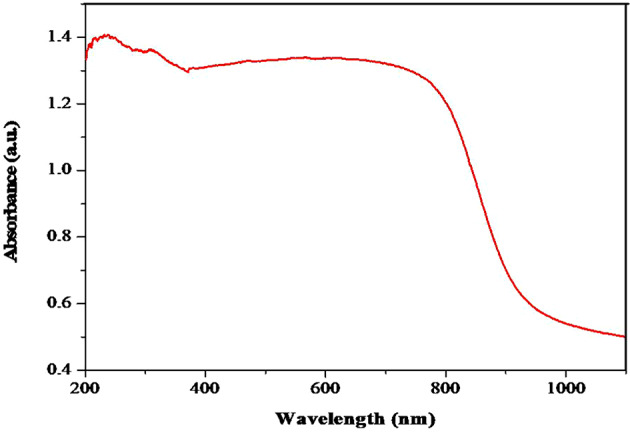
UV–Visible spectra of NiCoO_2_ nanocomposites

The optical investigations of NiCoO_2_ nanocomposites are shown in Fig. 3. The absorbance bands observed at 200 nm and 1100 nm correspond to Ni and Co, respectively**.** However, the absorbance spectrum of the NiCoO₂ nanocomposites exhibited broad absorption in the 400–800 nm visible region, showing a red shift compared with those of NiO and CoO [44].

#### Fluorescence

**Fig. 4:**
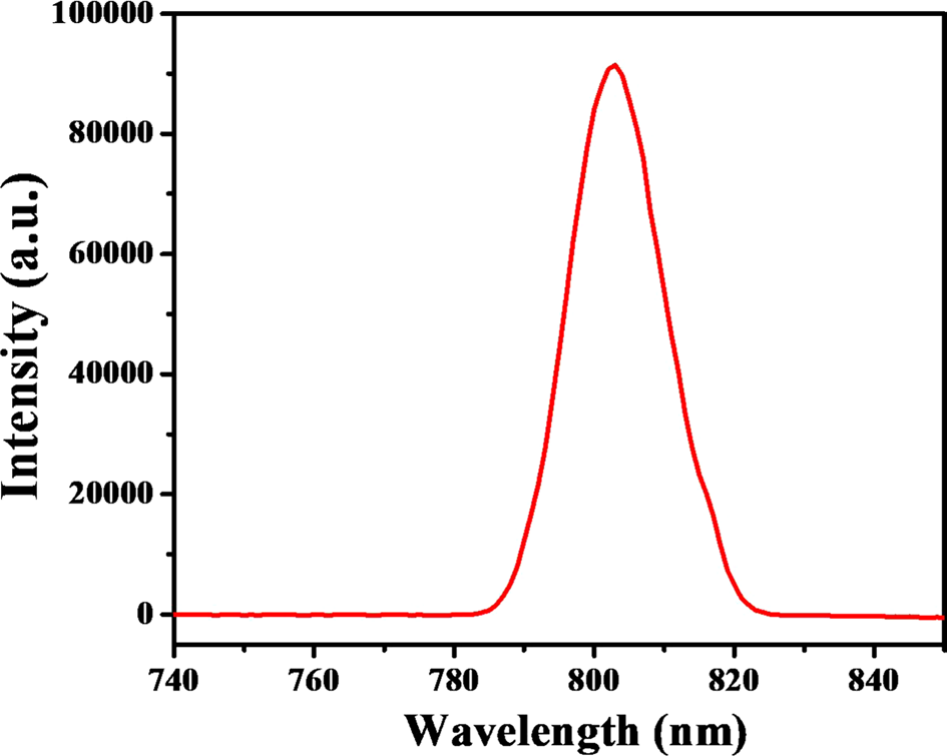
Fluorescence of NiCoO_2_ nanocomposite

Figure 4 illustrates the fluorescence spectrum of the NiCoO₂ nanocomposite measured in the wavelength range of 740–850 nm, which features a prominent emission peak at approximately 801 nm. The excitation of the sample was carried out at 530 nm in the visible region. The pronounced near-infrared emission around 801 nm arises from surface defect states. These defect-induced mid-gap levels enable electronic transitions that emit photons at longer wavelengths upon appropriate excitation, shifting the emission toward the red/near-infrared region [45].

#### FT-IR spectra

**Fig. 5:**
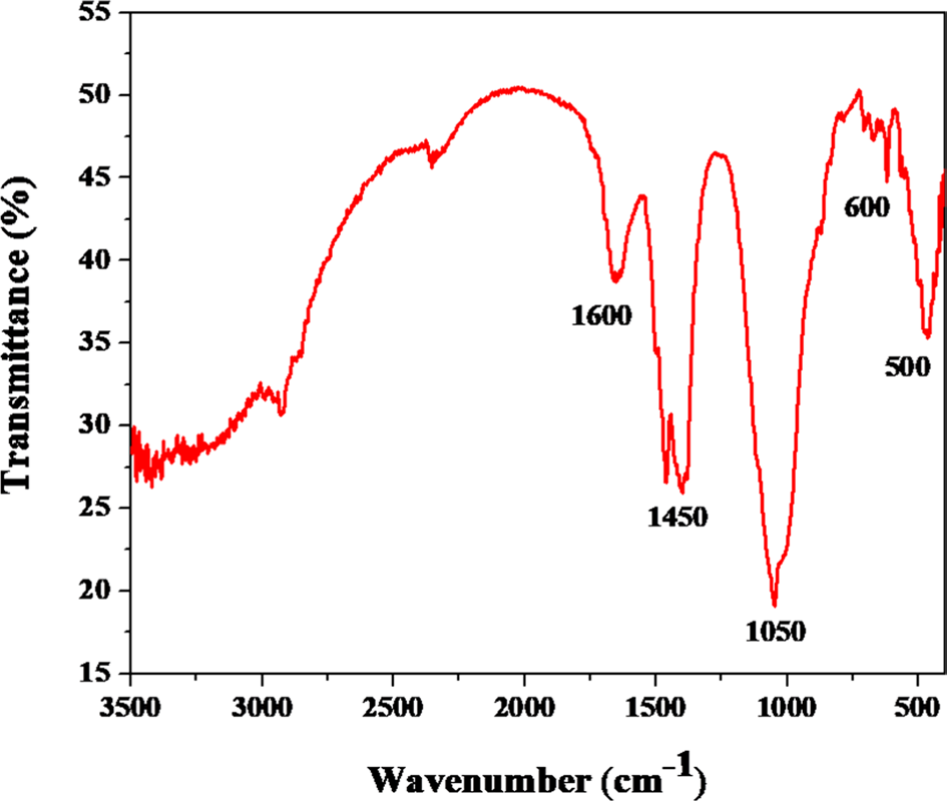
FT-IR spectrum of NiCoO_2_ nanocomposites

Figure 5 shows the FT-IR spectrum measured in the wavenumber range of 400 to 3600 cm^−1^ for the biosynthesized NiCoO₂ nanocomposites, exhibiting bands at 1450 cm^−1^, 600 cm^−1^, and 500 cm^−1^, which are attributed to C–O moieties from the precursors used in the reaction and to Co–O and Ni–O stretching vibrations, respectively. The FT-IR spectra of the NiCoO_2_ nanocomposites also suggest possible interactions between Ni and Co ions and the *M. oleifera* plant extract during the bioreduction process [46].

#### BET

**Fig. 6:**
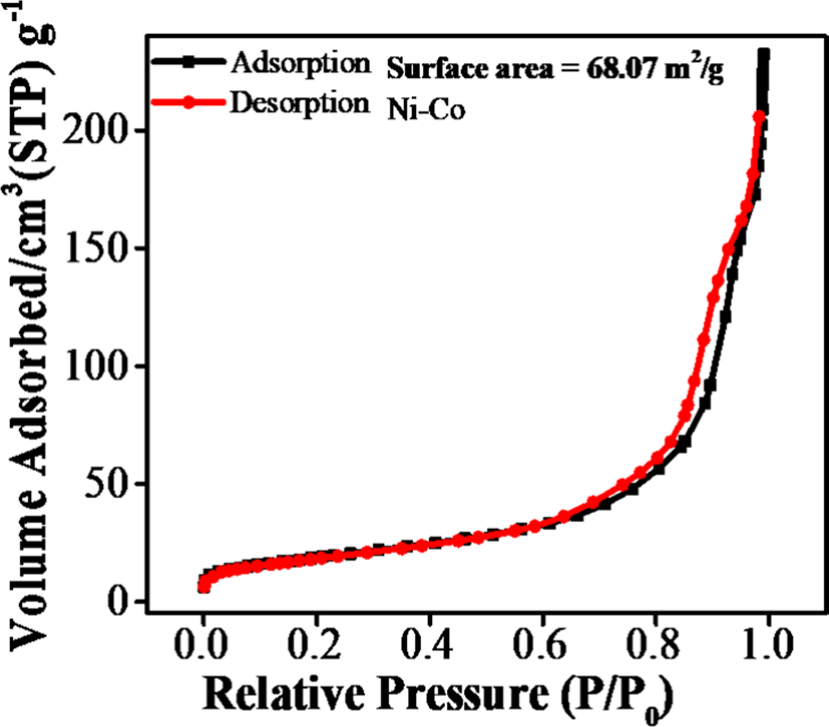
BET of NiCoO_2_ nanocomposite

Nitrogen adsorption–desorption experiments (Fig. 6) were performed to assess the surface area properties of the NiCoO₂ nanocomposite. The isotherms exhibited a Type IV hysteresis loop, indicative of a mesoporous structure. BET analysis yielded a specific surface area of 68.07 m^2^/g, while the Barrett–Joyner–Halenda (BJH) method confirmed the presence of mesopores based on the pore size distribution profile. These findings confirm that the synthesized NiCoO_2_ nanocomposite possesses a mesoporous morphology with a moderate surface area, which may influence its catalytic and adsorption performance. A larger specific surface area means that more atoms or molecules are exposed at the surface, offering more reactive sites for catalytic conversion, and mesopores provide optimal trade-offs, including high surface area, good accessibility, and fast mass transport, making them highly favorable for catalysis [43].

#### SEM and EDS

**Fig. 7:**
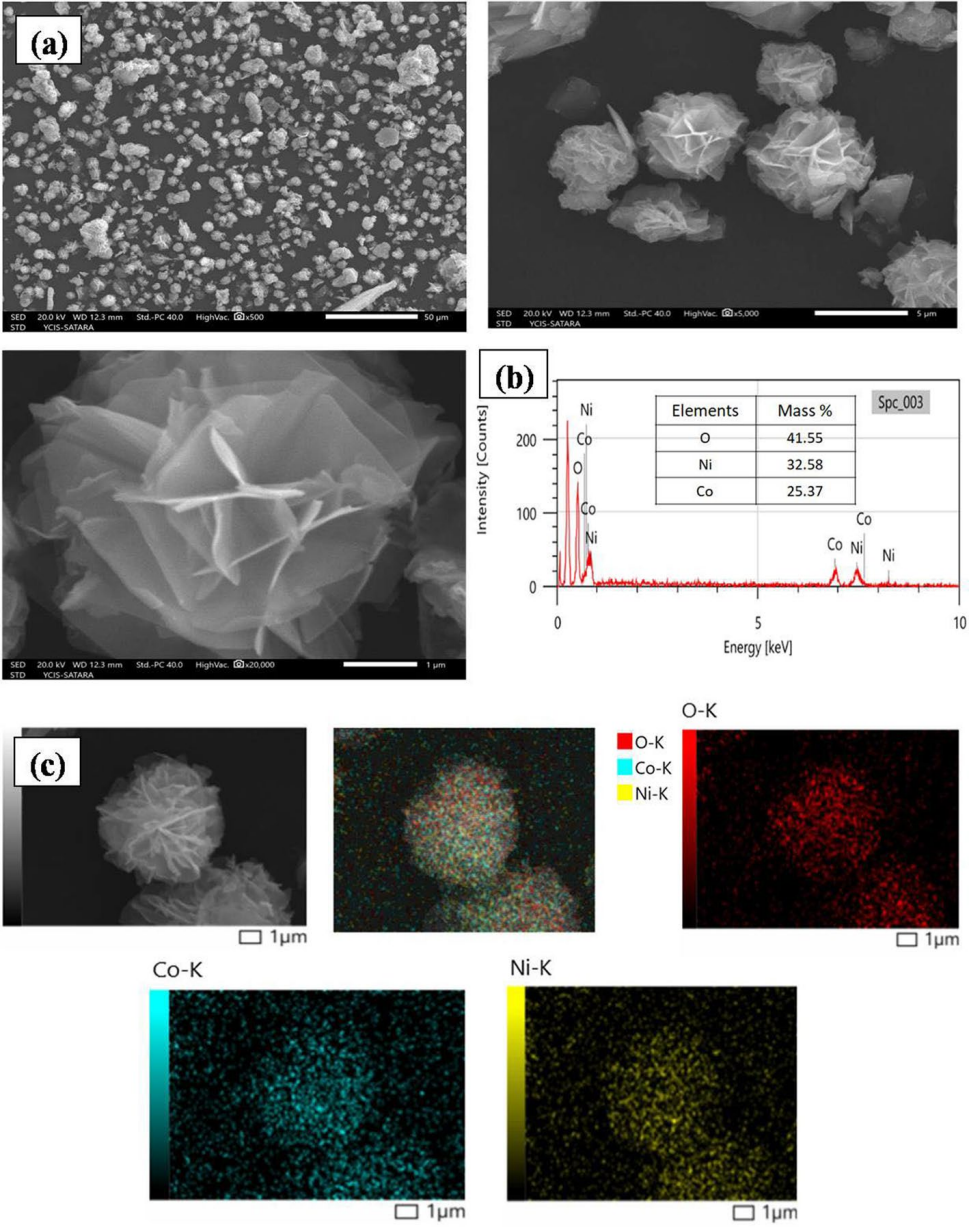
**a**: SEM images of the biosynthesized NiCoO₂ nanocomposite showing uniformly distributed, hierarchical flower-like nanostructures composed of thin, interconnected nanosheets; **b**: EDS of NiCoO_2_ nanocomposite and **c**: Elemental mapping of NiCoO_2_ nanocomposite

The SEM was used for the characterization of the surface morphology of the synthesized NiCoO₂ nanocomposite. It provides information about the morphology and surface structure of the nanoparticles, as shown in Fig. 7a. At low and high magnifications, the SEM images of the NiCoO₂ nanocomposite were observed to exhibit a spherical morphology. The average particle size of the nanocomposite was estimated to be approximately 54 nm, as determined from the SEM images. EDS is a powerful tool used for elemental analysis of the synthesized nanocomposite. From this analysis, it was confirmed that no other elemental impurities were present in the synthesized nanocomposite. The synthesized nanocomposite contained 25.87, 32.58, and 41.55 wt% of Co, Ni, and O, respectively, as shown in Fig. 7b at their corresponding energy values. The formation of the biosynthesized NiCoO₂ nanocomposite was further confirmed by the EDS spectrum [47]. The elemental mapping of NiCoO_2_, shown in Fig. 7c, provided elemental distribution images in which the distribution of nickel (Ni), cobalt (Co), and oxygen (O) was indicated by distinct color overlays (e.g., Ni-K, Co-K, and O-K). This type of mapping allows direct visualization of how each element is dispersed throughout the nanocomposite.

#### TEM

**Fig. 8:**
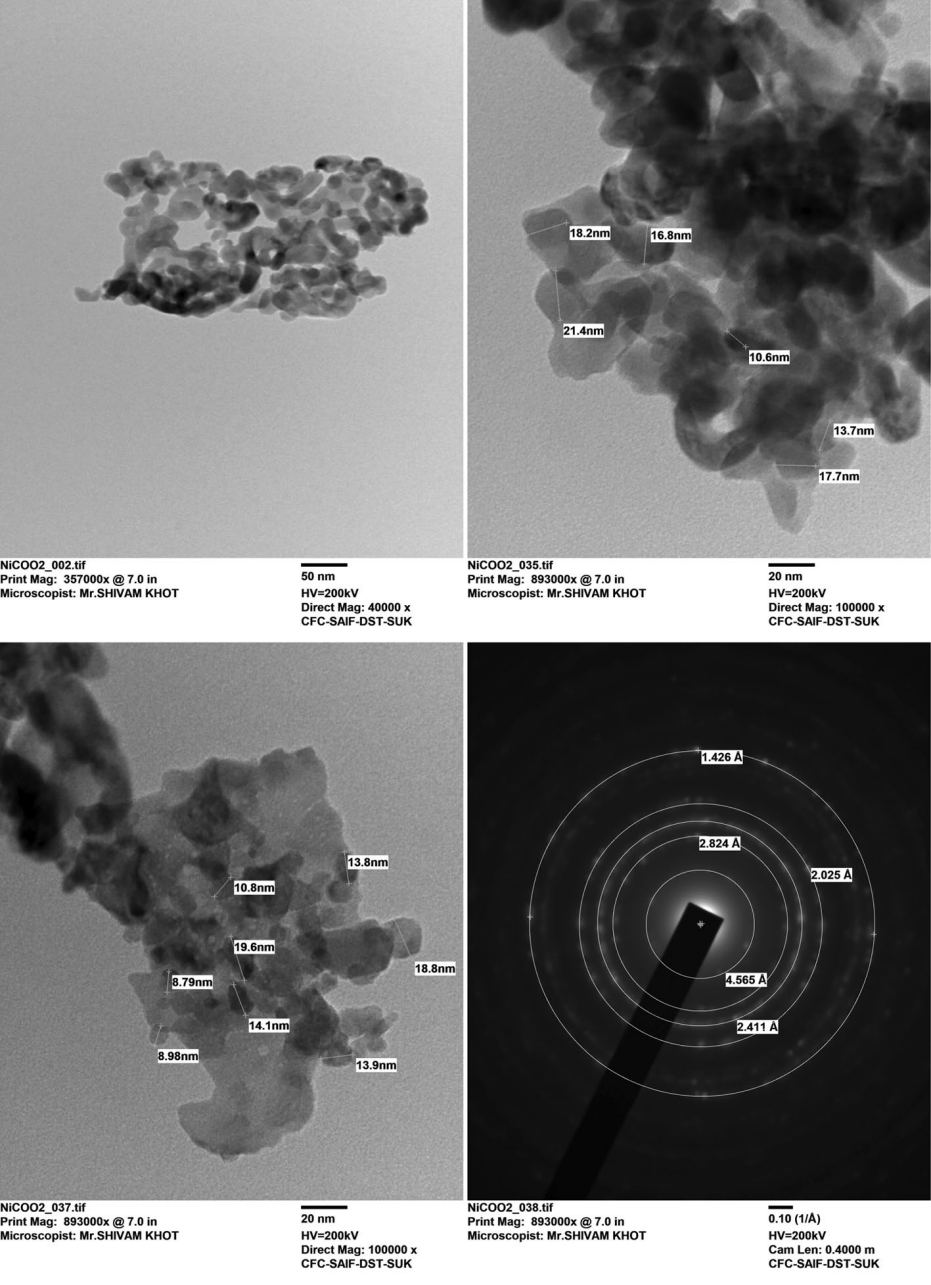
TEM images of the biosynthesized NiCoO₂ nanocomposite

The TEM analysis of the biosynthesized NiCoO₂ nanocomposite reveals the formation of aggregated nanosized particles with predominantly spherical to slightly irregular morphology (Fig. 8). Low- and high-magnification TEM images show that the nanocomposite consists of closely packed nanocrystallites, with particle sizes ranging from approximately 8.7 to 21.4 nm and an average size of about 15–18 nm, indicating effective control of nucleation and growth by the phytochemicals present in the *Moringa oleifera* leaf extract. The smaller particle size observed in TEM compared to SEM confirms that SEM reflects agglomerated secondary particles, whereas TEM provides information on the primary nanocrystallites. The selected area electron diffraction (SAED) pattern displays well-defined concentric rings, confirming the polycrystalline nature of the NiCoO₂ nanocomposite. The calculated interplanar spacings correspond well to the characteristic crystallographic planes of cubic NiCoO_2_ and are in good agreement with the XRD results, thereby confirming the successful formation of a crystalline NiCoO₂ nanocomposite with nanoscale dimensions suitable for enhanced catalytic and antimicrobial applications.

### Screening of catalyst

Table 1 summarizes the performance of various catalysts reported for the synthesis of pyrazolopyrano[2,3-d]pyrimidines. Among the catalysts evaluated, the biosynthesized NiCoO₂ nanocomposite exhibited high catalytic efficiency, achieving a 98% yield in just 20 min under ambient conditions in a 1:1 ethanol–water medium, compared with conventional catalysts such as ZnO, DABCO, and γ-Al₂O₃-based systems. The observed catalytic performance of NiCoO₂ can be attributed to its unique nanostructured morphology, high surface area, and the synergistic interaction between Ni and Co sites, which facilitate efficient adsorption and activation of reactants in the multicomponent condensation reaction. Moreover, unlike many previously reported catalysts, the NiCoO₂ nanocomposite is eco-friendly, easily recyclable over multiple cycles, and synthesized via a green, plant-mediated route, highlighting its potential and practical advantages as a sustainable heterogeneous catalyst for this transformation.

**Table 1:** Previously reported catalyst in the synthesis of pyrazolopyrano(2,3-d) pyrimidine

Sr. No	Catalyst	Solvent	Time(min)	Temperature (°C)	Yield (%)	References
1	Meglumine (20 mg)	EtOH/H₂O (1:1)	15	60	96%	[48]
2	DABCO(10 mol %)	EtOH	15	60	92	[49]
3	γ-Al₂O₃@silane-TMG(10–20 mg)	EtOH	60	60–90	98	[49]
4	Fe₃O₄@MCM-41@IL/Pd(0.10 mol %)	EtOH	15	50–80	98	[50]
5	SCMNPs@uridine/Zn(15–20 mg)	EtOH	30	50–80	97	[50]
6	Nano-Al₂O₃(20 mg)	EtOH	40	60–90	90	[26]
7	Piperidine(10–20 mol %)	EtOH	10	60	94	[26]
8	ZnO nanoparticles (20 mg)	EtOH	60	60–90	90	[26]
9	Imidazole (10–20 mol %)	EtOH	30	60	90	[26]
10	NiCoO_2_ nanocomposite (5 mol%)	EtOH:H_2_O (1:1)	20	40	98	Present Work

Table 2 represents the screening of the catalyst on the basis of different catalyst loadings (mol%) used in the synthesis of pyrazolopyrano[2,3-d]pyrimidine**.** From this catalyst screening, we conclude that 5 mol% of the catalyst gives the highest yield of the product within the shortest reaction time.

**Table 2:** Screening of Catalyst (mol%) optimizationin the synthesis of pyrazolopyrano(2,3-d) pyrimidine

Sr. No	Catalyst (mol%)	Time ( Min)	Yield (%)
1	0.5	120	70
2	1.0	100	75
3	1.5	90	80
4	2.0	80	83
5	3.0	70	88
6	3.5	60	90
7	4.0	50	92
8	4.5	30	95
9	5.0	20	98
10	5.5	20	98
11	6.0	20	98

### Screening of solvent

The solvent is essential for optimizing catalytic activity. The inorganic nature of the nanocomposite catalyst and the organic nature of the reactants were evaluated in various solvents, as shown in Table 3. The 1:1 ethanol–water mixture provided the highest yields. Ethanol, a polar solvent with both hydrophilic and hydrophobic properties, can dissolve a wide range of organic compounds, while water is particularly effective at dissolving ionic and polar substances. This combination dissolves both reactants more efficiently than other solvents, thus enhancing reaction efficiency.

**Table 3:** Previously reported solvents in the synthesis of pyrazolopyrano[2,3-d]pyrimidines

Sr. No	Solvent system	Time (min)	Temperature(°C)	Yield (%)	References
1	DES	15	60–80	80	[51]
2	Water	120	100	85	[51]
3	Solvent-free	2	50–80	92	[52]
4	Water:ethanol (1:1)	5	80–90	94	[53]
5	Water	40	100	94	[25]
6	Ethanol	60	78	80	[25]
7	MeCN	20	82	50	[54]
8	Ethanol–acetic acid	360	80–100	87	[55]
9	Methanol	5	65	80	[26]
10	DCM	60	65	82	[56]
11	EtOH:H_2_O (1:1)	20	40	98	Present work

### Synthesis different derivatives of pyrazolopyrano[2,3-d]pyrimidine

The presence of electron-withdrawing groups on the aldehyde enhances the electrophilicity of the carbonyl carbon, facilitating nucleophilic attack by barbituric acid. This increased reactivity results in a higher yield of the product than that obtained from aldehydes containing electron-donating groups, which reduce the electrophilicity of the carbonyl carbon and hinder nucleophilic attack.

#### Proposed mechanism of synthesis pyrazolopyrano[2,3-d]pyrimidines

The reaction mechanism begins with the base-catalyzed Knoevenagel condensation of barbituric acid with an aromatic aldehyde to form an α,β-unsaturated arylidene barbiturate intermediate. After that, condensation takes place between ethyl acetoacetate and hydrazine hydrate to form a hydrazone, which cyclizes through intramolecular nucleophilic attack of the remaining NH₂ group on the keto carbonyl group. This transformation yields pyrazolone. Then, this active pyrazolone undergoes Michael addition to the α,β-unsaturated arylidene barbiturate, resulting in the formation of a new C–C bond. Finally, the resulting Michael adduct undergoes cyclization to form the pyrazolopyrano[2,3-d]pyrimidine compound.

### Spectral data for the compound 1e, 4e & 7e

#### 3-methyl-4-phenyl-4,8-dihydropyrazolo[4’,3’:5,6]pyrano[2,3-d]pyrimidine- 5,7(1H,6H)-dione (1e)

*White solid: FTIR (KBr)*: 600 cm^−1^, 1100 cm^−1^, 1400 cm^−1^, 1590 cm^−1^, 2250 cm^−1^, 2900 cm^−1^, and 3400 cm^−1^. The FTIR spectrum of 3-methyl-4-phenyl-4,8-dihydropyrazolo[4’,3’:5,6]pyrano[2,3-d]pyrimidine-5,7(1H,6H)-dione showed characteristic absorptions at 3400 cm⁻1 (N–H stretching), 2900 cm⁻1 (aliphatic C–H), 2250 cm⁻1 (conjugated unsaturation), 1590 cm⁻1 (C = C/C = N), 1400 cm⁻1 (C–N), 1100 cm⁻1 (C–O–C of pyran ring), and 600 cm⁻1 (ring deformation). These bands confirm the presence of the expected functional groups in the synthesized compound.

^**1**^**H NMR (400 MHz, DMSO)**: δ11.21 (s, 1H, NH), 10.12(s, 2H, NH), 7.18 (t, J = 6.8 Hz, 2H, Ar–H), 7.13(d, *J* = 6.8 Hz, 2H, Ar–H), 6.90(t, J = 6.8 Hz 1H, Ar–H), 5.41 (s, 1H, CH), and 2.18(s, 3H, CH_3_).

The ^1^H NMR spectrum was recorded at 400 MHz in DMSO and showed a singlet at δ 11.21 ppm corresponding to one NH proton, while a second singlet at δ 10.12 ppm was assigned to two NH protons. The aromatic region exhibited signals at δ 7.18 ppm as a triplet (J = 6.8 Hz, 2H), δ 7.13 ppm as a doublet (J = 6.8 Hz, 2H), and δ 6.90 ppm as a triplet (J = 6.8 Hz, 1H), attributable to the phenyl ring protons. A singlet at δ 5.41 ppm corresponded to the methine proton, and a singlet at δ 2.18 ppm was assigned to the methyl group.

^**13**^**C NMR (101 MHz, DMSO)** δ170.09, 161.07, 152.98, 151.15, 145.57, 142.33, 128.30, 127.08, 125.46, 104.99, 92.62, 32.57, and 10.61.

The ^13^C NMR spectrum, recorded at 101 MHz in DMSO, displayed signals at δ 170.09, 161.07, 152.98, 151.15, 145.57, 142.33, 128.30, 127.08, 125.46, 104.99, 92.62, 32.57, and 10.61 ppm, consistent with the expected carbonyl, heteroaromatic, aromatic, methine, and methyl carbons of the proposed structure. These complete spectral details support the assigned structure of the synthesized compound.

**Mass spectrum**: The mass spectrum of 3-methyl-4-phenyl-4,8-dihydropyrazolo[4’,3’:5,6]pyrano[2,3- d]pyrimidine-5,7(1H,6H)-dione shows a molecular ion peak at m/z ≈ 360**,** confirming the expected molecular weight of the compound. The presence of a single dominant peak with negligible fragmentation indicates a highly stable fused heterocyclic framework with resistance to breakdown under ionization conditions (Table 4).

**Table 4:**
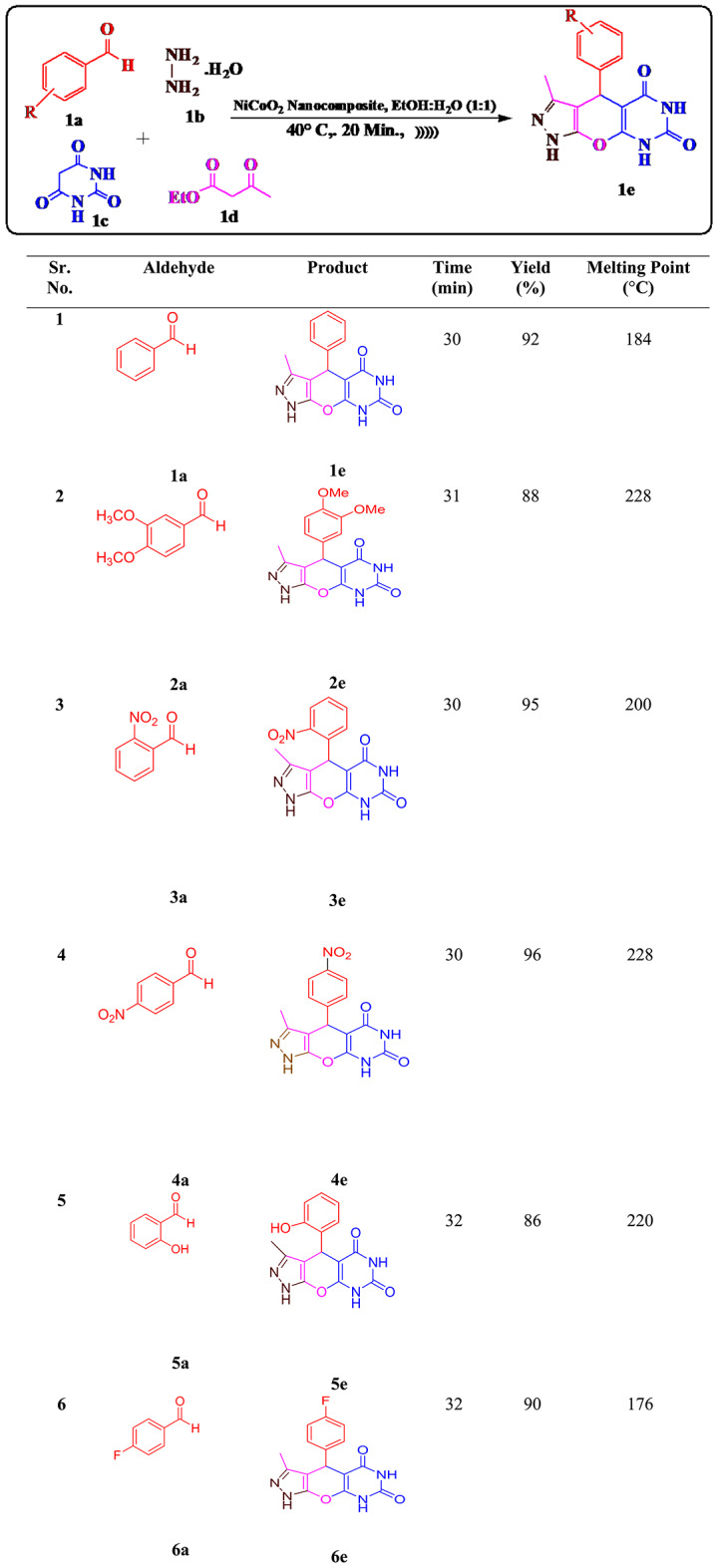

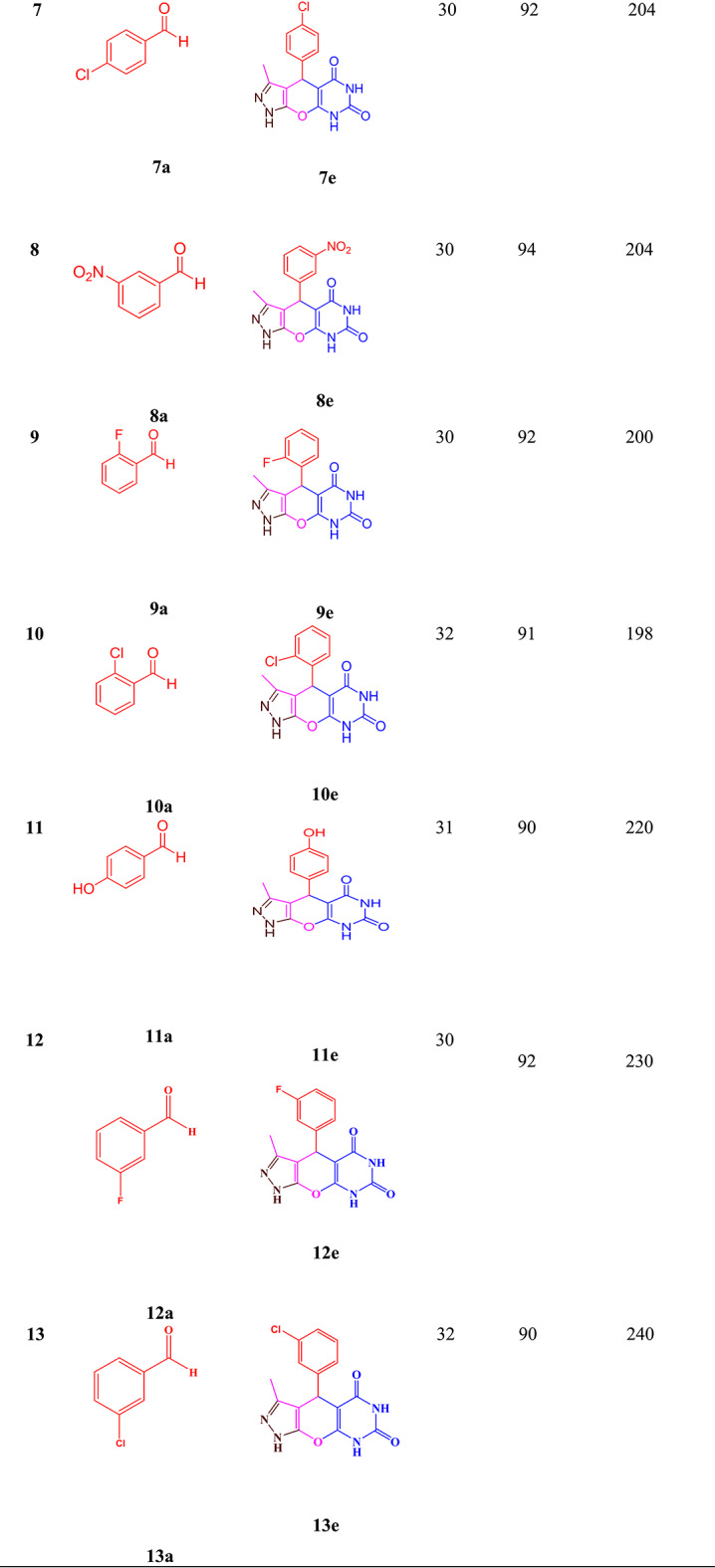
Synthesis different derivatives of Pyrazolopyrano[2,3-d]pyrimidine from various aldehydes reacted with hydrazine hydrate, barbituric acid and ethyl acetoacetatein Ethanol: water (1:1)solvent system

#### 3-Methyl-4-(4-nitrophenyl)-4,8-dihydropyrazolo[3′,4′:5,6]Pyrano[2,3-d]pyrimidine 5,7(1H,6H)-dione (4e):

**FTIR (KBr)**: 600 cm^−1^, 1100 cm^−1^, 1400 cm^−1^, 1600 cm^−1^, 2900 cm^−1^, and 3400 cm^−1^.

The FTIR spectrum of 3-Methyl-4-(4-nitrophenyl)-4,8-dihydropyrazolo[3′,4′:5,6]pyrano[2,3-d]pyrimidine-5,7(1H,6H)-dione showed characteristic bands at 3400 cm⁻1 (N–H), 2900 cm⁻1 (C–H), 1600 cm⁻1 (C = C/C = N), 1400 cm⁻1 (C–N), 1100 cm⁻1 (C–O–C of pyran ring), and 600 cm⁻1 (ring deformation). These absorptions confirm the presence of the expected functional groups in the compound.

^**1**^**H NMR (400 MHz, DMSO) δ**11.16 (s, 1H, NH), 10.11 (s, 2H, NH), 8.04 (d, J = 8 Hz, 2H, Ar–H), 7.34 (d, J = 7.6 Hz, 2H, Ar–H), 5.52(s, 1H, CH), and 2.23(s, 3H, CH_3_).

The ^1^H NMR spectrum was recorded at 400 MHz in DMSO and showed a singlet at δ 11.16 ppm corresponding to one NH proton, along with a singlet at δ 10.11 ppm assigned to two NH protons. The aromatic protons appeared as doublets at δ 8.04 ppm (J = 8.0 Hz, 2H) and δ 7.34 ppm (J = 7.6 Hz, 2H), consistent with substituted aromatic ring protons. A singlet at δ 5.52 ppm was attributed to the methine proton, while the singlet at δ 2.23 ppm corresponded to the methyl group.

^**13**^**C NMR (101 MHz, DMSO)** δ165.40, 160.33, 155.11, 151.55, 151.16, 145.90, 143.67, 128.23, 123.12,104.11, 91.12, 31.60, and 10.43.

The ^13^C NMR spectrum, recorded at 101 MHz in DMSO, exhibited signals at δ 165.40, 160.33, 155.11, 151.55, 151.16, 145.90, 143.67, 128.23, 123.12, 104.11, 91.12, 31.60, and 10.43 ppm, which are in agreement with the expected carbonyl, heteroaromatic, aromatic, methine, and methyl carbons of the proposed structure. These complete spectral data support the structural assignment of the synthesized compound.

**Mass spectrum:** The mass spectrum of 3-methyl-4-(4-nitrophenyl)-4,8-dihydropyrazolo[3′,4′:5,6]pyrano[2,3- d]pyrimidine-5,7(1H,6H)-dione shows a prominent molecular ion peak at m/z ≈ 341**,** confirming the molecular weight of the compound. The presence of a single intense peak with minimal fragmentation indicates a highly stable fused heterocyclic structure, with little breakdown under ionization conditions.

#### 4-(4-chlorophenyl)-3methyl-4,8-dihydropyrazolo[3′,4′:5,6]Pyrano[2,3- d]pyrimidine5,7(1H,6H)-dione (7e):

**FTIR (KBr)**: 600 cm^−1^, 1100 cm^−1^, 1400 cm^−1^, 1600 cm^−1^, 2100 cm^−1^, 2900 cm^−1^, and 3400 cm^−1^.

The FT-IR spectrum (KBr) of 4-(4-chlorophenyl)-3-methyl-4,8-dihydropyrazolo[3′,4′:5,6]pyrano[2,3-d]pyrimidine-5,7(1H,6H)-dione confirms the expected functional groups. The band at 3400 cm⁻^1^ is assigned to N–H stretching, while 2900 cm⁻^1^ corresponds to aliphatic C–H stretching. The absorption at 1600 cm⁻^1^ indicates C = O stretching of the dione group, and 1400 cm⁻^1^ is due to aromatic C = C stretching. The band at 1100 cm⁻^1^ is attributed to C–O–C stretching of the pyran ring, whereas the peak at 600 cm⁻^1^ confirms C–Cl stretching. These signals support the proposed molecular structure.

^**1**^**H NMR (400 MHz, DMSO)** δ12.65 (s, 1H, NH), 10.12 (s, 2H, NH), 7.91 (d, J = 8 Hz, 2H, Ar–H), 7.05 (d, J = 7.2 Hz, 2H, Ar–H), 5.41(s, 1H, CH), and 2.23 (s, 3H, CH_3_).

The 1H NMR spectrum (400 MHz, DMSO) supports the proposed structure. The singlet at δ 12.65 ppm (1H) and 10.12 ppm (2H) are assigned to NH protons, confirming nitrogen-bound hydrogen in the heterocyclic system. Two aromatic doublets at δ 7.91 ppm (2H, J = 8.0 Hz) and 7.05 ppm (2H, J = 7.2 Hz) indicate a para-substituted aromatic ring. The singlet at δ 5.41 ppm (1H) corresponds to a methine proton, while the doublet at δ 2.23 ppm (3H, J = 12.2 Hz) is assigned to a methyl group. These signals collectively confirm the expected molecular framework.

^**13**^**C NMR (101 MHz, DMSO)** δ165.87, 160.99, 154.27, 151.69, 151.20, 143.10, 140.97, 128.16, 127.53, 104.62, 91.48, 32.09, and 14.35.

The 13C NMR spectrum (101 MHz, DMSO) shows thirteen distinct carbon signals, consistent with the proposed structure. The downfield signals at δ 165.87–151.20 ppm are assigned to carbonyl and heterocyclic carbons attached to electronegative atoms. Peaks at δ 143.10 and 140.97 ppm correspond to quaternary carbons, while δ 128.16 and 127.53 ppm are attributed to aromatic carbons. Signals at δ 104.62 and 91.48 ppm indicate fused ring carbons, whereas δ 32.09 and 14.35 ppm are assigned to methine and methyl carbons, respectively. These data support the expected molecular framework.

**Mass spectrum**: The mass spectrum of 4-(4-chlorophenyl)-3-methyl-4,8-dihydropyrazolo[3′,4′:5,6]pyrano[2,3- d]pyrimidine-5,7(1H,6H)-dione shows a molecular ion peak at m/z ≈ 354, confirming the expected molecular weight of the compound. A prominent fragment peak at m/z ≈ 331 corresponds to the loss of a small neutral fragment (such as HCl or NH₃), while the limited fragmentation pattern indicates the high stability of the fused heterocyclic system.

### Atom economy & atom efficiency

**Fig. 9:**
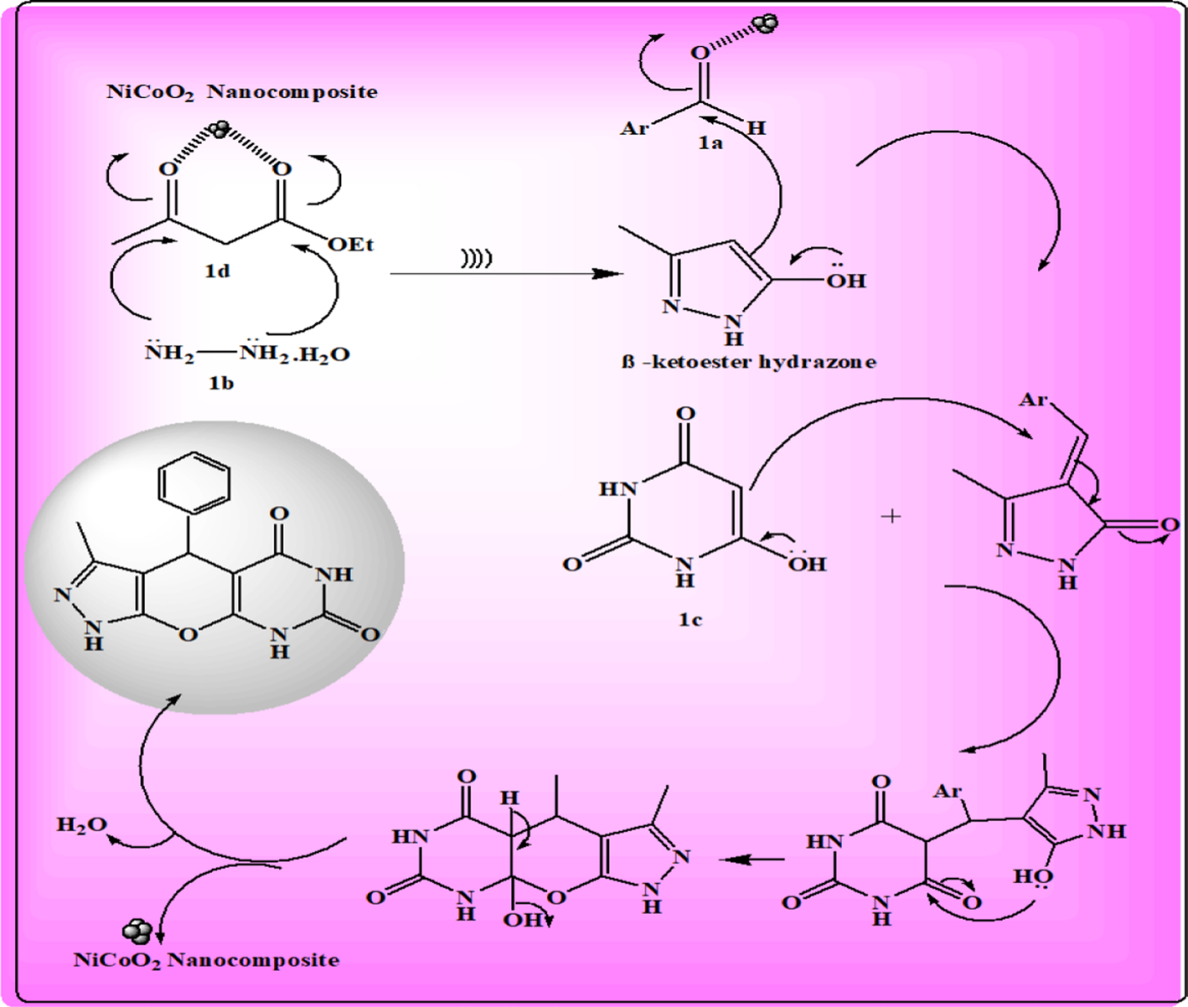
Plausible mechanism for synthesis of pyrazolopyrano[2,3-d]pyrimidines

The above Table 5 shows the atom economy values for a number of synthesized products, labelled 1e to 11e. Atom economy and atom efficiency are key metrics in green chemistry, representing the efficiency with which reactants are converted into the desired products. Higher atom economy values indicate a more ecological and efficient process with minimal waste generation. Atom economy reflects the theoretical incorporation of reactant atoms into the final product, whereas atom efficiency considers the actual experimental yield and practical utilization of materials [57]. The atom economy values for the desired products range from 73.9% to 76.5%, and the atom efficiency values range from 70.6% to 74.1%, demonstrating that the synthetic methodology employed is relatively efficient across all products. Notably, products 3e, 4e, and 8e exhibit the highest atom economy due to minimal formation of by-products and more effective incorporation of all reactant fragments into the desired molecular framework, which is consistent with their higher isolated yields and cleaner reaction profiles. This observation aligns with the principles of green chemistry, where reactions with fewer elimination steps and solvent-free or one-pot pathways typically show higher atom utilization [58]. Product 2e shows the highest atom efficiency of 74.1%, which signifies highly efficient utilization of the starting materials in this specific reaction, as shown in Fig. 9.

**Table 5:** Atom economy and atom efficiency of desired product

Product name	1e	2e	3e	4e	5e	6e	7e	8e	9e	10e	11e
Atom economy	73.9	77.1	76.5	76.5	75.0	74.1	75.8	76.5	74.1	75.8	75.0
Atom efficiency	70.6	74.1	73.4	73.4	71.8	70.9	72.7	73.4	70.9	72.7	71.8

### Turnover number and turnover frequency

**Fig. 10:**
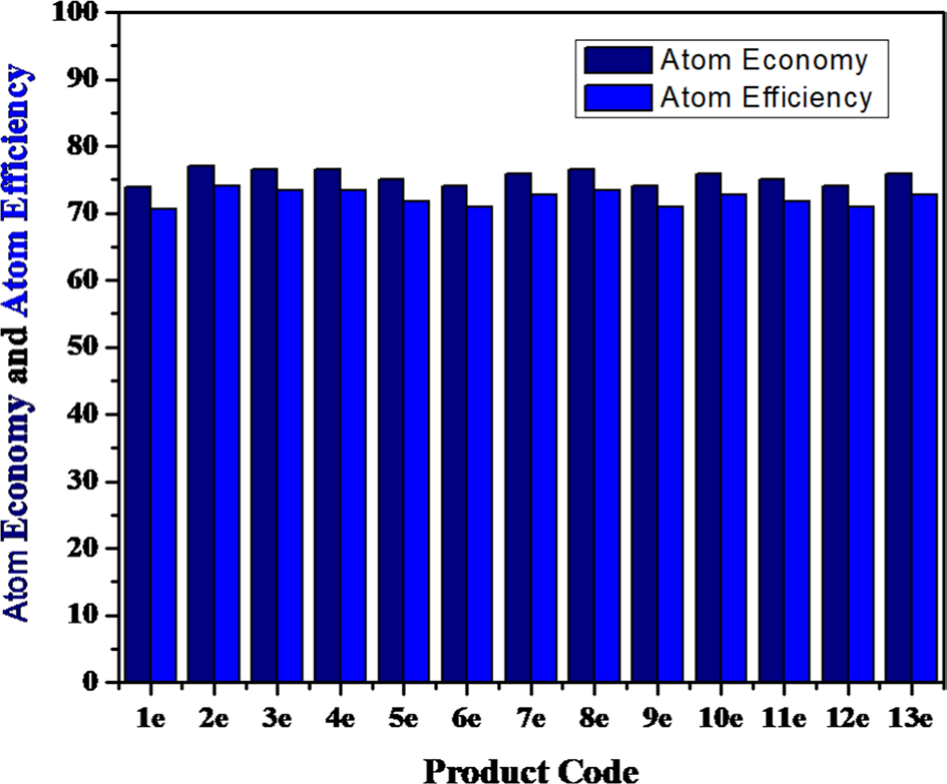
Atom economy and atom efficiency of desired product

The NiCoO₂ nanocomposite exhibited high catalytic performance in the synthesis of pyrazolopyrano[2,3-d]pyrimidine, as evidenced by the Turnover Number (TON) and Turnover Frequency (TOF) data summarized in Table 6. The observed TON values ranged from 11,466 to 12,800, indicating the catalyst’s ability to undergo thousands of catalytic cycles before deactivation. Correspondingly, the TOF values ranged from 21,513 to 25,600 h^−1^, demonstrating the rapid catalytic activity of the nanocomposite. Notably, the NiCoO₂ nanocomposite achieved its peak performance, with a TOF of 25,600 h^−1^, and a TON of 12,800 during the synthesis of product 4e (Fig. 10). The higher TON and TOF observed for product 4e can be attributed to its favorable electronic and structural features, which enhance substrate–catalyst interactions and accelerate reaction kinetics, leading to more efficient catalytic cycles. Similar substituent-dependent variations in turnover values have been reported in heterogeneous catalytic systems, where electron-donating or activating groups promote faster conversion rates [59]. These results underscore the critical influence of optimized reaction conditions and substrate–catalyst compatibility in maximizing catalytic efficiency. Overall, the study demonstrates that the NiCoO₂ nanocomposite serves as an effective and sustainable heterogeneous catalyst for organic transformations, aligning with the principles of green chemistry.

**Table 6:** TON and TOF values of synthesized NiCoO_2_Catalyst using green method

Product	1e	2e	3e	4e	5e	6e	7e
TON	12,266	11,733	12,666	12,800	11,466	12,000	12,266
TOF (hr^−1^)	24,533	23,006	25,333	25,600	21,513	22,514	24,533

Product	8e	9e	10e	11e	12e	13e
TON	12,533	12,266	12,133	12,000	12,266	12,133
TOF (hr^−1^)	25,066	24,533	22,764	23,529	24,533	22,764

### Antimicrobial study of NiCoO_2_ nanocomposite

**Fig. 11:**
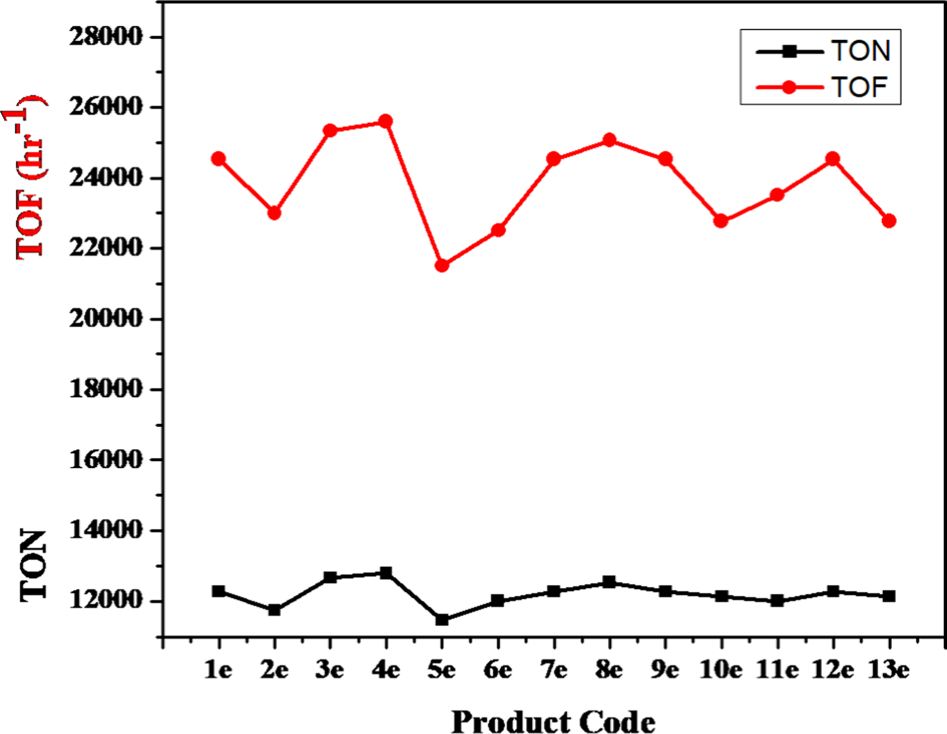
TON and TOF of desired products

**Fig. 12:**
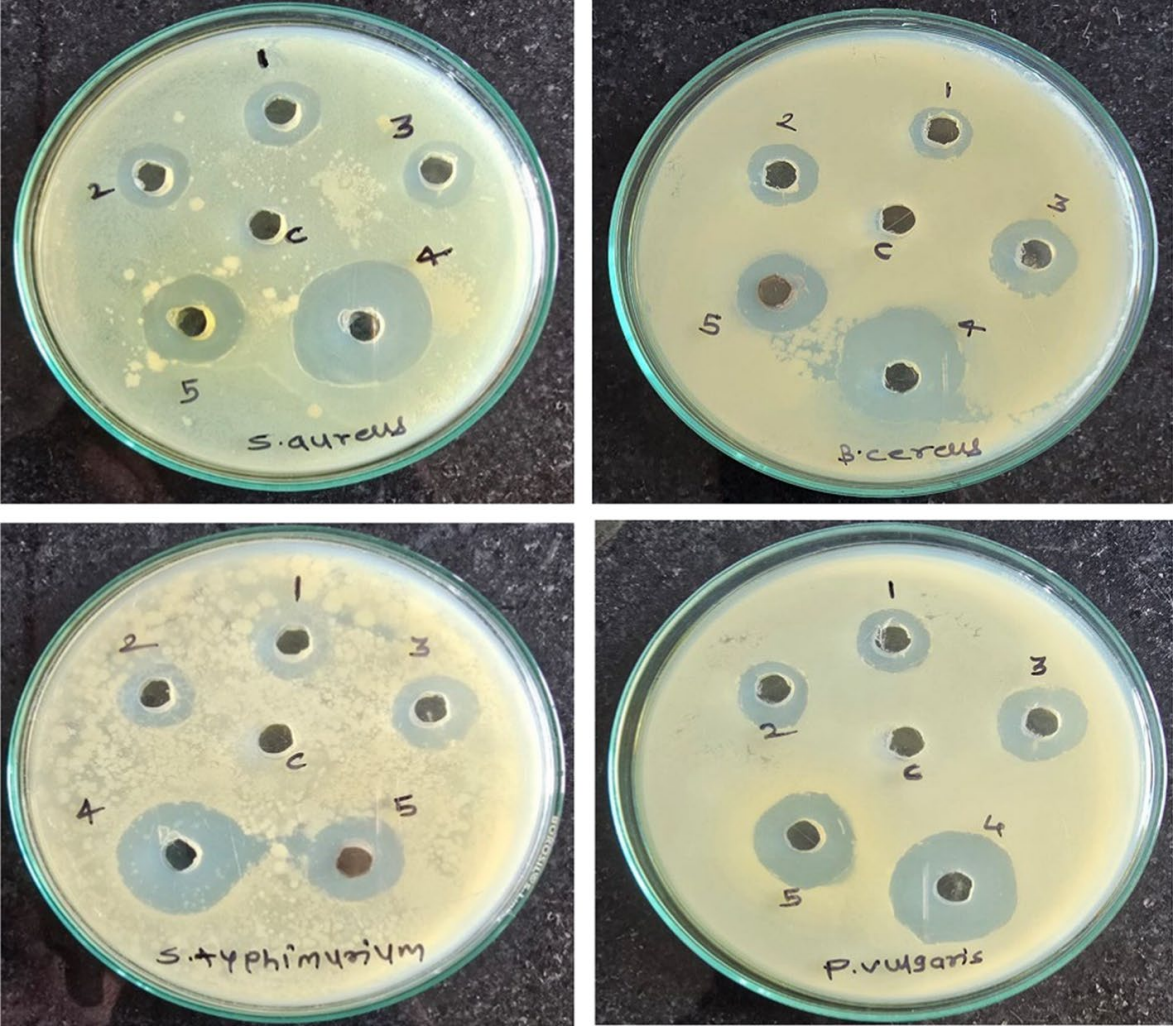
Agar well diffusion assay showing zones of inhibition against S. aureus, B. cereus, P.vulgaris, and S. typhimurium for (1) Ni NPs, (2) CoO₂, (3) plant extract, (4) NiCoO₂ nanocomposite, (5) antibiotic (streptomycin), and control (sterile distilled water). Data are presented as mean ± SD (n = 3). Statistical analysis was performed using one-way ANOVA. Differences were considered statistically significant at p < 0.05

**Fig. 13:**
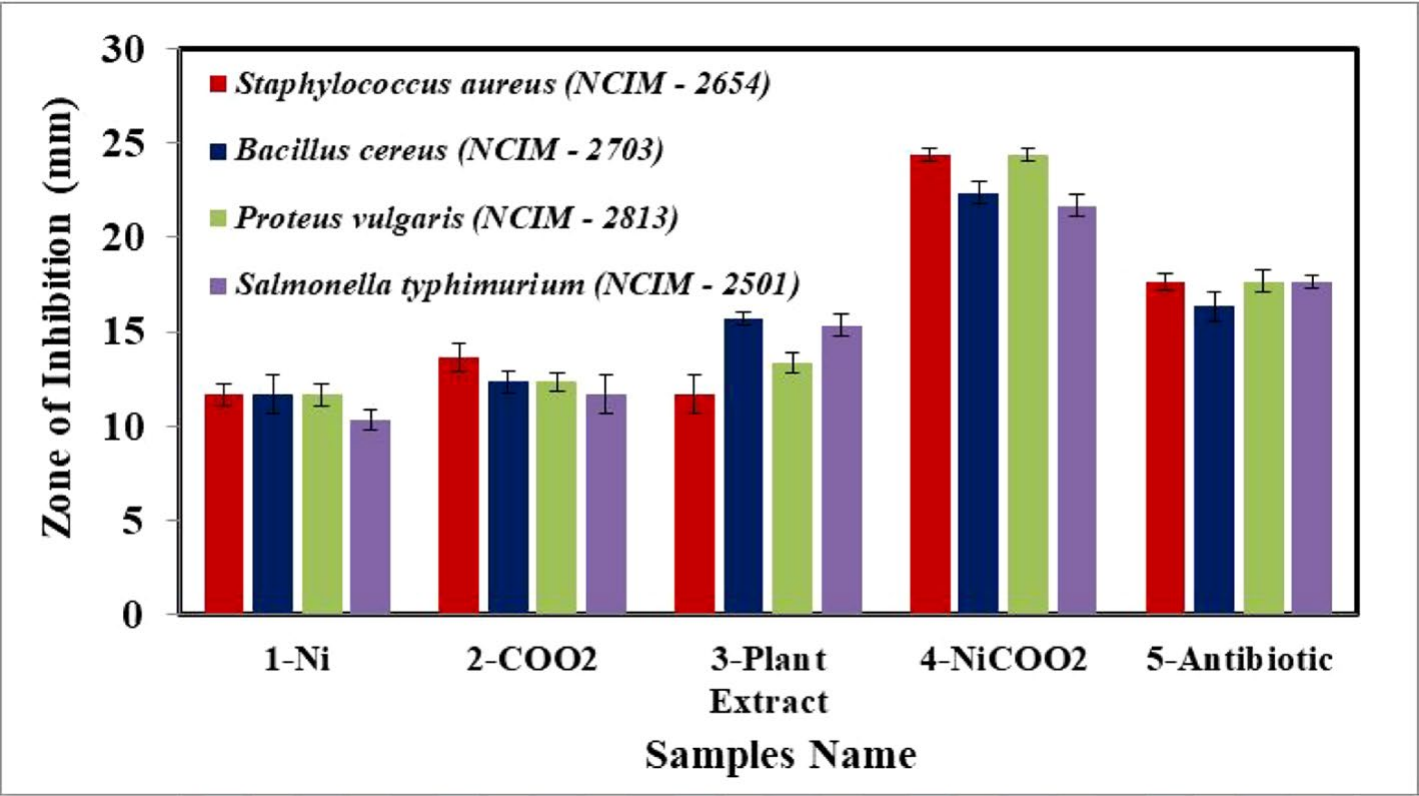
Statistical comparison of antimicrobial activity of Ni NPs, CoO₂, plant extract, NiCoO₂ nanocomposite, and antibiotic against selected bacterial strains. Data are expressed as mean ± SD (n = 3). Statistical significance was evaluated using one-way ANOVA (p < 0.05). The symbol (*) indicates a statistically significant difference compared to the control

The NiCoO₂ nanocomposite exhibited antibacterial activity, which may be attributed to the generation of reactive oxygen species (ROS) that induce oxidative stress on the bacterial cell membrane, ultimately leading to bacterial cell death [60]. Similar findings have been reported by Hu and co-workers, who demonstrated that NiCoO₂-based metal oxides influence charge-transfer reactions, in which surface oxygen or moisture is responsible for generating ROS that contribute to the inhibition of microorganisms [61]. The antimicrobial efficacy of the materials was evaluated using sterile distilled water as a negative control. The agar well diffusion method was used to assess antimicrobial activity against selected bacterial strains, namely *S. aureus, B. cereus, P. vulgaris****,*** and *S. typhimurium*, with streptomycin serving as the standard reference antibiotic. The synthesized materials demonstrated clear zones of inhibition, indicating their effectiveness in suppressing the growth of all tested pathogens. The results suggest that the synthesized NiCoO₂ nanocomposite exhibits moderate antimicrobial performance compared with the standard antibiotic, previously reported nanomaterials, and plant-based extracts (Figs. 11, 12 and 13)**.** Data are presented as the mean ± standard error (SE) of three independent replicates (n = 3) [62].

#### Statistical analysis

All experimental data were subjected to basic pre-processing prior to statistical evaluation. Data sets were screened for consistency, and no outliers were excluded. Results obtained from antimicrobial assays were recorded from three independent experiments (n = 3) and are expressed as mean ± standard deviation (SD). Statistical significance among different samples was evaluated using one-way analysis of variance (ANOVA). A probability value of p < 0.05 was considered statistically significant. All statistical analyses were performed using GraphPad Prism software (version 9.0; GraphPad Software, San Diego, CA, USA).

#### Antimicrobial study of synthesized product

The antimicrobial activity of the chemically synthesized 13 derivatives was evaluated using dimethyl sulfoxide (DMSO) as a negative control. The antimicrobial analysis was performed using the agar well diffusion method against the bacterial strains *S. aureus and B. cereus* (Gram-positive), and *S. typhimurium and P. vulgaris* (Gram-negative). These antibacterial studies revealed that the synthesized derivatives exhibited inhibitory activity against the growth of *S. aureus, B. cereus, S. typhimurium,* and *P. vulgaris*, which are associated with nosocomial infections. The present study confirmed that most of the synthesized derivatives exhibited good antimicrobial activity, whereas some derivatives showed negligible activity against the tested pathogenic strains. The above data represent the mean ± standard error (SE) of three independent replicates (n = 3).

The previously reported study on pyrazolopyrano[2,3-d]pyrimidines showed good antimicrobial activity against both Gram-positive and Gram-negative bacterial strains, which was attributed to their ability to permeate the bacterial cell membrane [63]. In contrast, the present study demonstrated that the synthesized 13 derivatives exhibited good antimicrobial activity against both Gram-positive (*S. aureus and B. cereus*) and Gram-negative (*S. typhimurium and P. vulgaris*) bacteria using the agar well diffusion method. The presence of electron-donating groups may contribute to enhanced antibacterial activity. The present work further demonstrated that comparatively better activity against Gram-negative strains was observed than that reported previously, although a few derivatives showed negligible effects. Overall, while both studies confirm good antibacterial activity against Gram-positive bacteria, the present study indicates a broader antibacterial spectrum, likely due to structural variations among the synthesized derivatives (Tables 7 and 8).

**Table 7:** Zone of inhibition of1-Ni NPs, 2-CoO_2_, 3-Plant extract, 4-NiCoO_2_, 5-Antibiotic and C-Control Antimicrobial activity against human pathogens

Entry	Antibacterial study			
	Gram + Ve		Gram -Ve	
	*Staphylococcus aureus (NCIM-2654)*	*Bacillus cereus (NCIM—2703)*	*Proteus vulgaris (NCIM—2813)*	*Salmonella typhimurium* (NCIM—2501)
1-Ni	11.66 ± 0.57	11.66 ± 1.00	11.66 ± 0.57	10.33 ± 0.57
2-CoO_2_	13.66 ± 0.75	12.33 ± 0.57	12.33 ± 0.45	11.66 ± 1.00
3-Plant Extract	11.66 ± 1.00	15.66 ± 0.35	13.33 ± 0.57	15.33 ± 0.55
4-NiCoO_2_	24.33 ± 0.35	22.33 ± 0.57	24.33 ± 0.35	21.66 ± 0.57
5-Antibiotic	17.66 ± 0.45	16.33 ± 0.75	17.66 ± 0.57	17.66 ± 0.35

**Table 8:** Binding affinity comparison of compounds (1–11) and reference drug (12)

Protein	Compound	Binding energy	Reference Drug binding energy value (ΔG is shown in minus kcal/mol)
			Indomethacin (12)
4COX	Compound 1	-8.3	− 7.7
	Compound 2	-8.8	
	Compound 3	-8.5	
	Compound 4	-8.9	
	Compound 5	-8.6	
	Compound 6	-8.5	
	Compound 7	-8.6	
	Compound 8	-9.2	
	Compound 9	-8.6	
	Compound 10	-8.2	
	Compound 11	-8.2	

#### Molecular docking

Molecular docking was performed using the synthesized compounds and the reference compound to determine their binding affinities toward the target protein. The results of the molecular docking studies are presented in Table 9.

**Table 9:** Binding affinity and interactions of compounds (1–11) and indomethacin (12) against Cyclooxygenase 2

Entry	Cyclooxygenase-2 (4COX)			
	Binding affinity	Amino acid residue involved	Binding distance	Type of interactions
Compound 1	− 8.3	Lys83	4.71	π-alkyl
		Pro86	5.38	π-alkyl
		Arg120	4.80	π-cation
		Leu123	4.72	π-alkyl
		Phe470	2.35	Conventional H bond
		Glu524	3.79	π-anion
Compound 2	− 8..8	Lys83	4.86	π-alkyl
		Pro86	5.14	π-alkyl
		Val89	4.46	Alkyl
		Val116	4.14	Alkyl
		Arg120	3.21, 4.82	Conventional H bond, π-cation
		Leu123	4.60	π-alkyl
		Phe470	2.12	Conventional H bond
		Glu524	3.80	π-anion
Compound 3	− 8.5	Lys83	4.78	π-alkyl
		Pro86	4.69	π-alkyl
		Val89	4.34	π-alkyl
		Tyr115	2.70	Conventional H bond
		Arg120	2.98, 4.66, 4.83	Conventional H bond, π-cation, π-cation
		Leu123	4.88	π-alkyl
		Phe470	1.79	Conventional H bond
		Ser471	3.76	C-H bond
		Glu524	3.59	π-anion
Compound 4	− 8.9	Lys83	4.82	π-alkyl
		Pro86	4.79	π-alkyl
		Val89	4.66	π-alkyl
		Arg120	4.74	π-cation
		Leu123	4.83	π-alkyl
		Tyr355	2.67	Conventional H bond
		Phe470	2.04	Conventional H bond
		Glu524	3.61	π-anion
Compound 5	− 8.6	Lys83	5.06, 5.39	π-alkyl, π-alkyl
		Tyr115	3.04	Conventional H bond
		Arg120	3.01	Conventional H bond
		Tyr122	2.52, 4.88	Conventional H bond, π-π T shaped
		Leu123	4.68	π-alkyl
		Phe470	2.27	Conventional H bond
		Glu524	2.85	Conventional H bond
Compound 6	− 8.5	Lys83	4.82	π-alkyl
		Pro86	4.95	π-alkyl
		Val89	5.13	π-alkyl
		Arg120	4.77	π-cation
		Leu123	4.67	π-alkyl
		Phe470	2.15	Conventional H bond
		Glu524	3.77	π-anion
Compound 7	− 8.6	Lys83	4.73	π-alkyl
		Pro86	5.38	π-alkyl
		Val116	4.90	Alkyl
		Arg120	4.80	π-cation
		Leu123	4.72	π-alkyl
		Phe470	2.36	Conventional H bond
		Glu524	3.80	π-anion
Compound 8	− 9.2	Lys83	4.82	π-alkyl
		Pro86	3.78, 4.96	C-H bond, π-alkyl
		Val89	4.97	π-alkyl
		Arg120	3.11, 4.75	Conventional H bond, π-cation
		Leu123	4.72	π-alkyl
		Tyr355	3.30	Conventional H bond
		Phe470	2.09	Conventional H bond
		Glu524	3.71	π-anion
Compound 9	− 8.6	Lys83	5.09, 5.42	π-alkyl, π-alkyl
		Tyr115	2.99	Conventional fluorine
		Arg120	3.01	Conventional H bond
		Tyr122	2.45, 4.89	Conventional H bond, π-π T shaped
		Leu123	4.65	π-alkyl
		Phe470	2.29	Conventional H bond
		Glu524	2.75	Conventional H bond
Compound 10	− 8.2	Pro86	2.77	C-H bond
		Val89	4.91	π-alkyl
		His90	3.68	C-H bond
		Tyr115	5.67	π-π stacked
		Val116	5.09	π-alkyl
		Ser119	3.13	Conventional H bond
		Arg120	3.79	π-cation
		Tyr355	1.89	Conventional H bond
		Arg513	2.81	Conventional H bond
		Glu524	3.55, 3.70	π-anion, π-anion
Compound 11	− 8.2	Pro84	5.23	π-alkyl
		Val89	3.79, 4.30	π-sigma,π-alkyl
		Tyr115	3.78	π-donor
		Arg120	2.84, 4.11	Conventional H bond, π-cation
		Tyr355	2.77	Conventional H bond
		Ser471	3.32	C-H bond
		Glu524	4.38	π-anion
Indomethacin (12)	− 7.7	Lys83	4.33	Alkyl
		Val89	3.73, 4.82	π-sigma, π-alkyl
		His90	4.67	π-alkyl
		Tyr115	3.00	Conventional H bond
		Ser119	3.06	Conventional H bond
		Arg120	2.80, 4.48, 4.90	Conventional H bond, π-cation, π-cation
		Tyr355	3.28	C-H bond
		Val523	4.72	Alkyl
		Glu524	4.78	π-anion

**Fig. 14:**
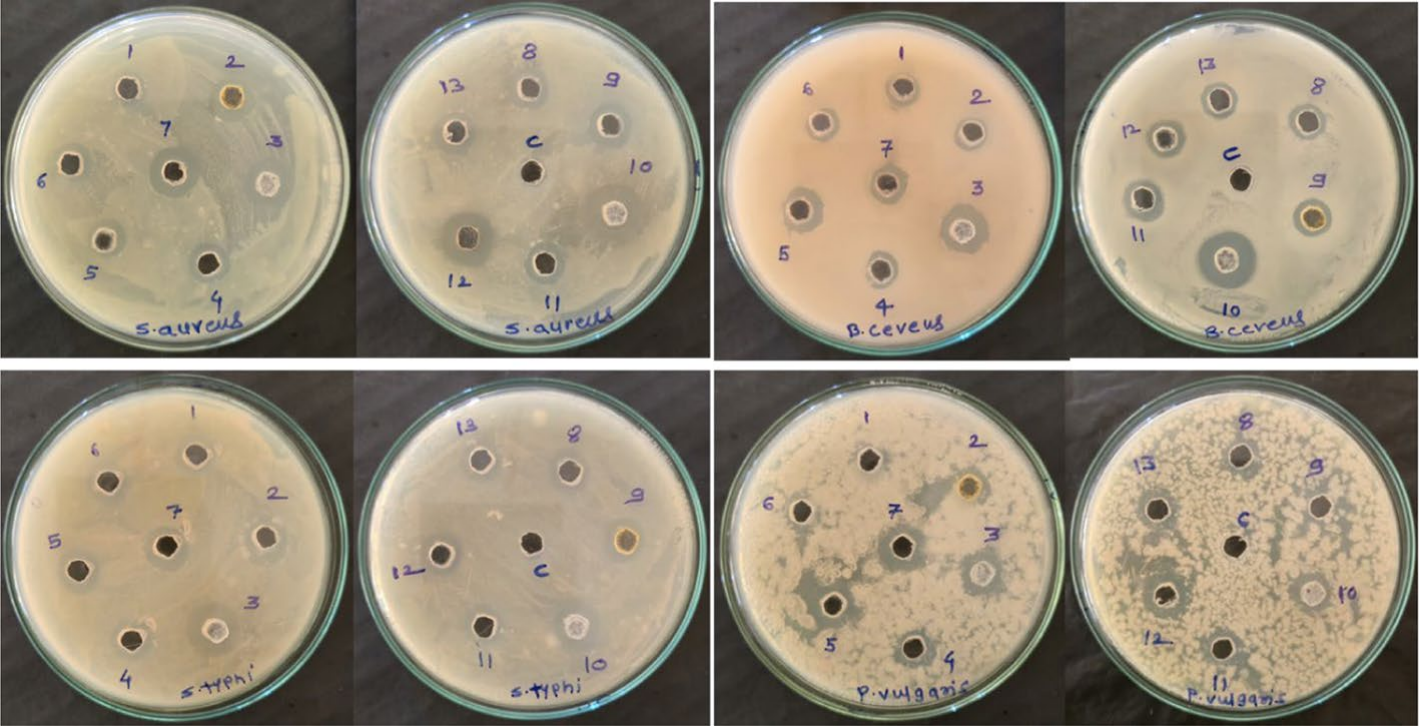
Antimicrobial activity of 1 to 13 various derivative samples and C-Control antimicrobial activity on the Staphylococcus aureus (NCIM -2654), Bacillus cereus (NCIM 2703), Salmonella typhimurium (NCIM-2501) and Proteus vulgaris (NCIM—2813) with zone of inhibition

**Fig. 15:**
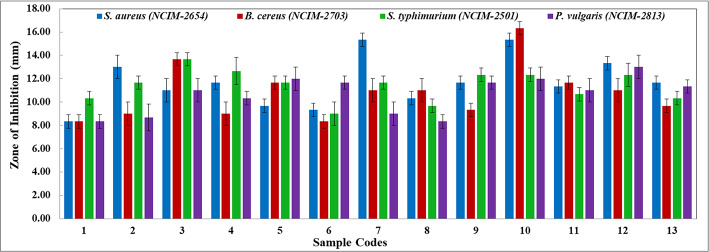
Zone of inhibition of 1 to 13 various derivative samples and C-Control antimicrobial activity against human pathogenic strains

**Fig. 16:**
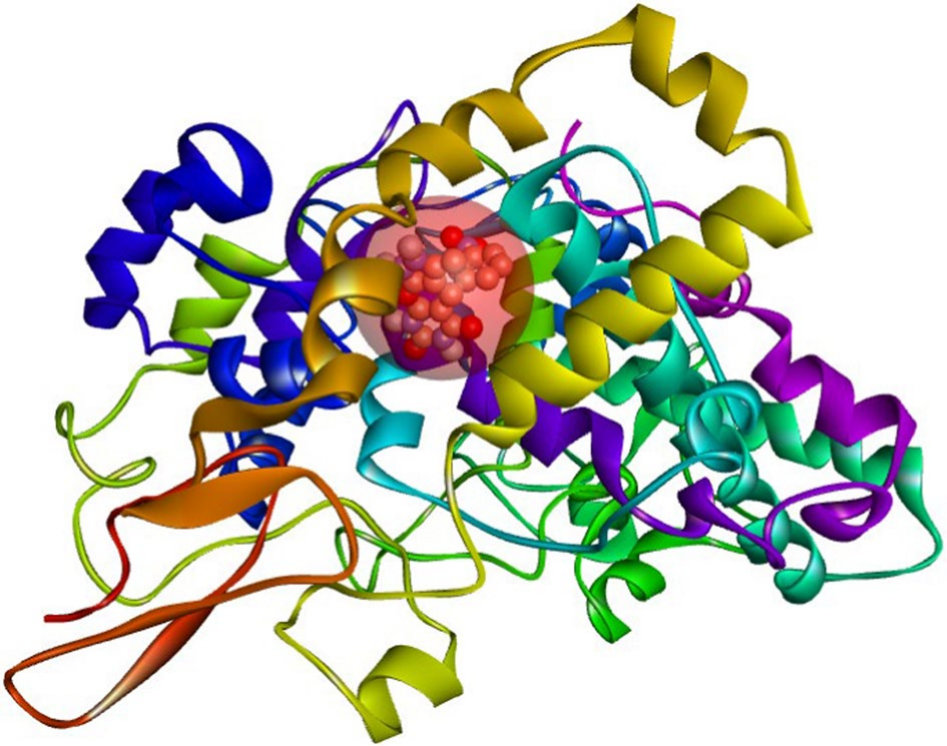
Molecular docking of compound 8 in the ribbon structure of 4COX

In this research, we evaluated compounds (1–12) as potential anti-inflammatory agents (Fig. 13). The docking results for binding to 4COX showed binding energies of − 8.3, − 8.8, − 8.5, − 8.9, − 8.6, − 8.5, − 8.6, − 9.2, − 8.6, − 8.2, − 8.2, and − 7.7 kcal/mol for compounds 1–12, respectively (Table 8; Figs. 14 and 15 and 16). The docking analyses demonstrated that compounds 1–12 exhibited different binding affinities toward the target protein. Based on their binding affinity (ΔG), the compounds can be ranked as follows: Compound 8 > Compound 4 > Compound 2 > Compound 5 = Compound 7 = Compound 9 > Compound 3 = Compound 6 > Compound 1 > Compound 10 = Compound 11 > Compound 12.

Compound 1 exhibited a binding energy of − 8.3 kcal/mol with 4COX and interacted with Lys83, Pro86, Arg120, Leu123, Phe470, and Glu524 amino acid residues. Compound 2 exhibited a binding energy of − 8.8 kcal/mol and interacted with Lys83, Pro86, Val89, Val116, Arg120, Leu123, Phe470, and Glu524 residues. Compound 3 showed a binding energy of − 8.5 kcal/mol and formed interactions with Lys83, Pro86, Val89, Tyr115, Arg120, Leu123, Phe470, Ser471, and Glu524 residues. Compound 4 exhibited a binding energy of − 8.9 kcal/mol through interactions with Lys83, Pro86, Val89, Arg120, Leu123, Tyr355, Phe470, and Glu524 residues.

Compound 5 exhibited a binding energy of − 8.6 kcal/mol and interacted with Lys83, Tyr115, Arg120, Tyr122, Leu123, Phe470, and Glu524 residues. Compound 6 displayed a binding energy of − 8.5 kcal/mol through interactions with Lys83, Pro86, Val89, Arg120, Leu123, Phe470, and Glu524 residues. The docking study of compound 7 revealed interactions with Lys83, Pro86, Val116, Arg120, Leu123, Phe470, and Glu524 residues, resulting in a binding energy of − 8.6 kcal/mol. Compound 8 exhibited the highest binding affinity (− 9.2 kcal/mol) and interacted with Lys83, Pro86, Val89, Arg120, Leu123, Tyr355, Phe470, and Glu524 residues. Compound 9 displayed a binding energy of − 8.6 kcal/mol through interactions with Lys83, Tyr115, Arg120, Tyr122, Leu123, Phe470, and Glu524 residues.

Compound 10 exhibited a binding energy of − 8.2 kcal/mol and interacted with Pro86, Val89, His90, Tyr115, Val116, Ser119, Arg120, Tyr355, Arg513, and Glu524 residues. Similarly, compound 11 displayed a binding energy of − 8.2 kcal/mol through interactions with Pro84, Val89, Tyr115, Arg120, Tyr355, Ser471, and Glu524 residues. The docking study of indomethacin (compound 12) revealed interactions with Lys83, Val89, His90, Tyr115, Ser119, Arg120, Tyr355, Val523, and Glu524 residues, with a binding energy of − 7.7 kcal/mol.

#### Recyclability of catalyst

**Fig. 17:**
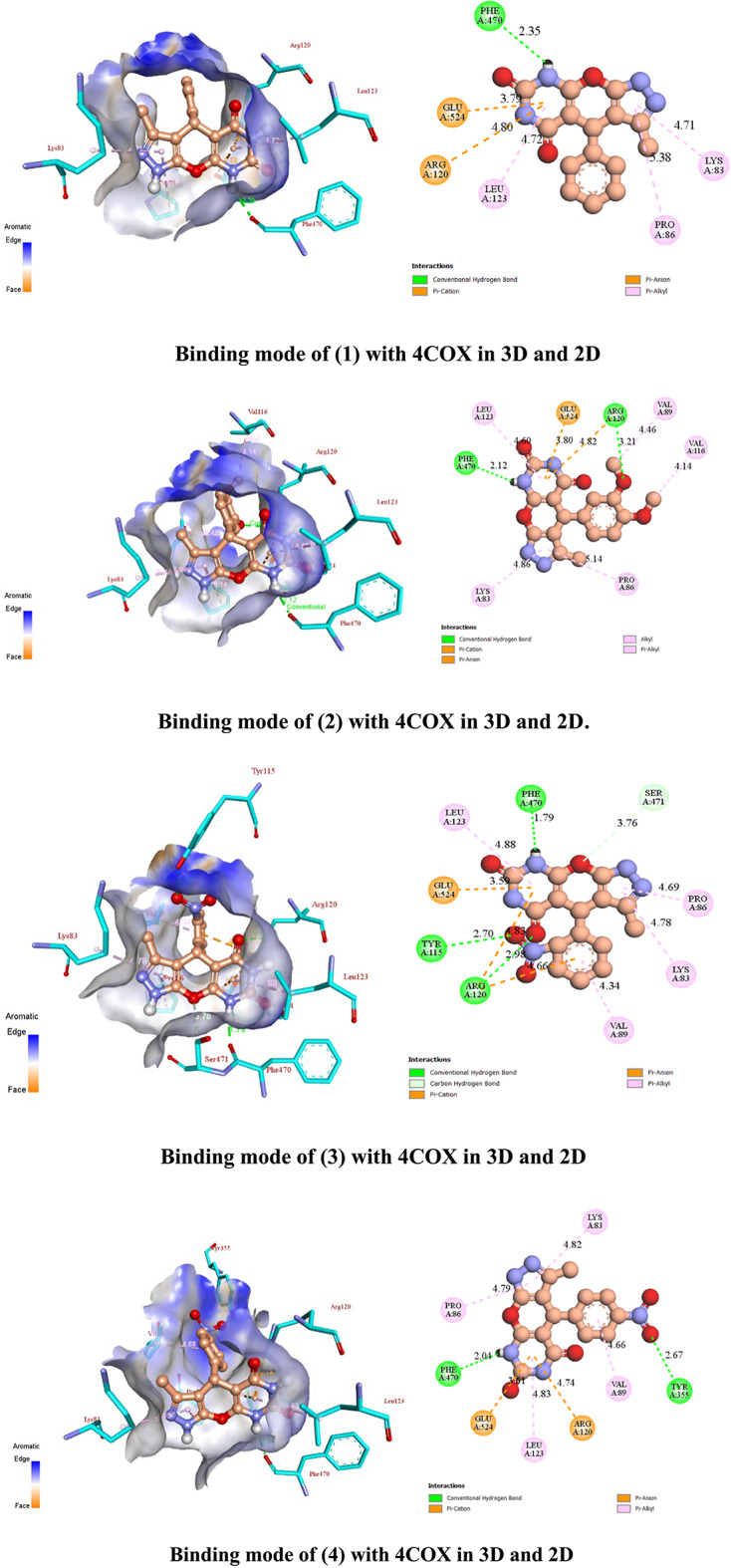

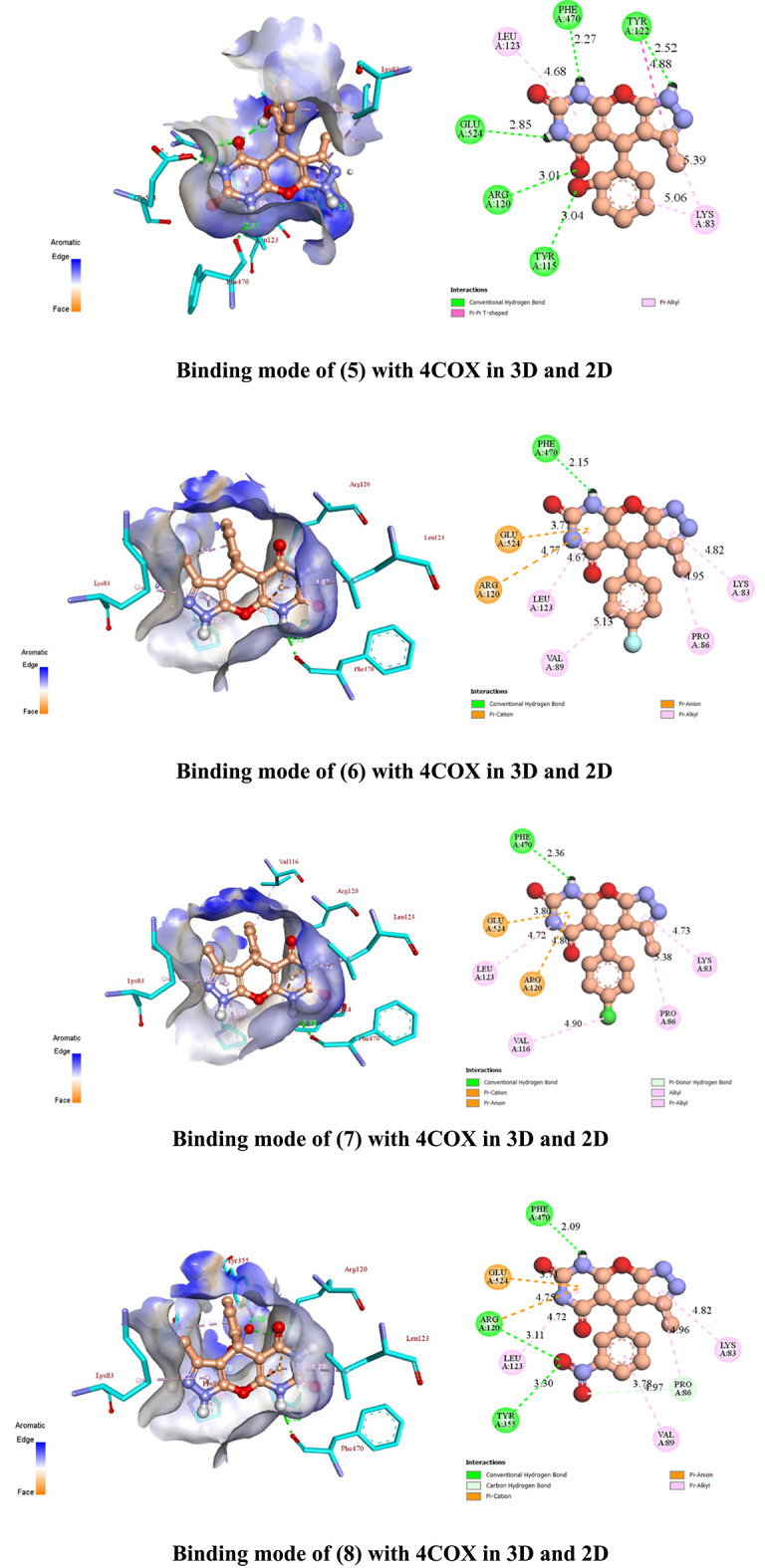

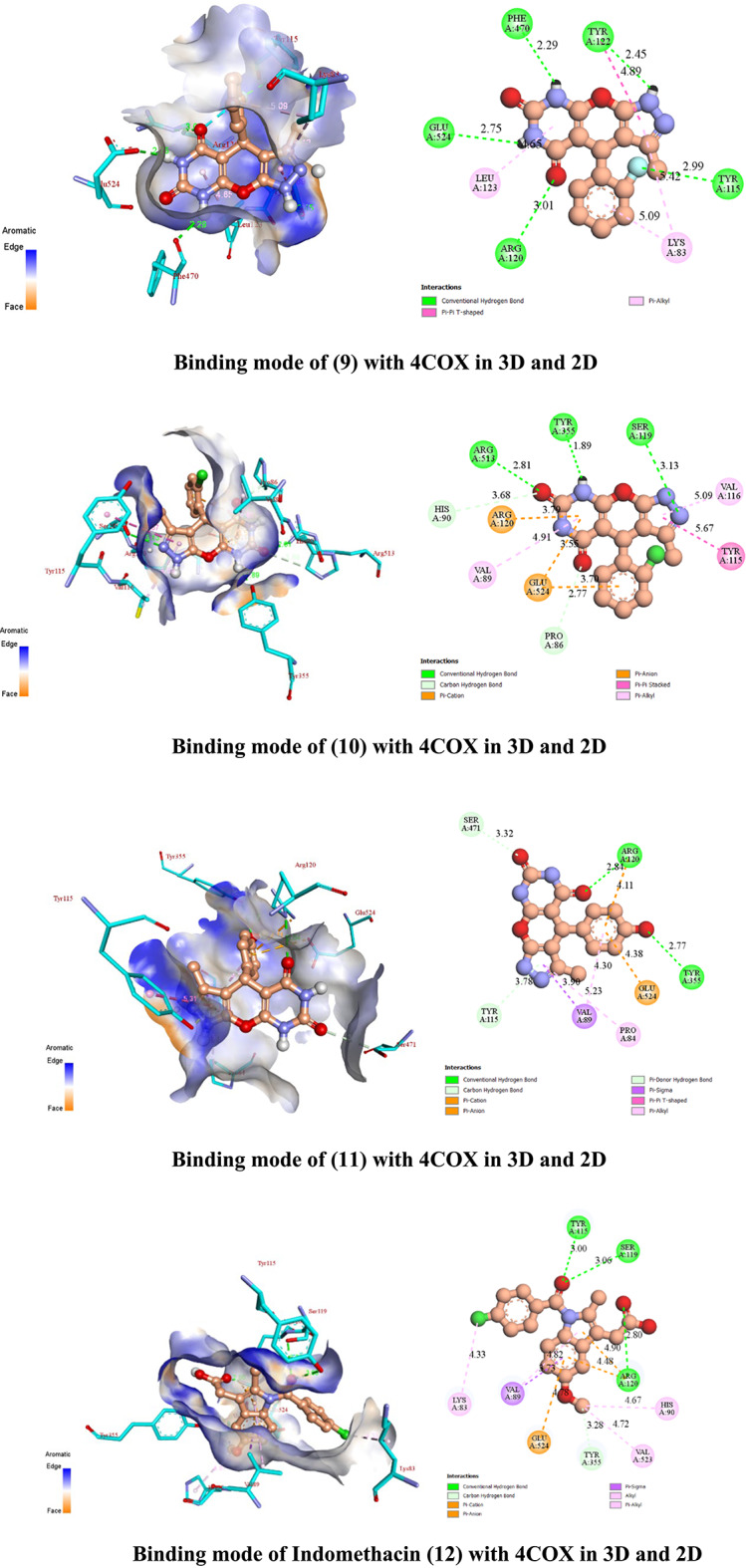
Binding interaction of compounds 1–12 within the binding pocket 4COX protein 3D and 2D

**Fig. 18:**
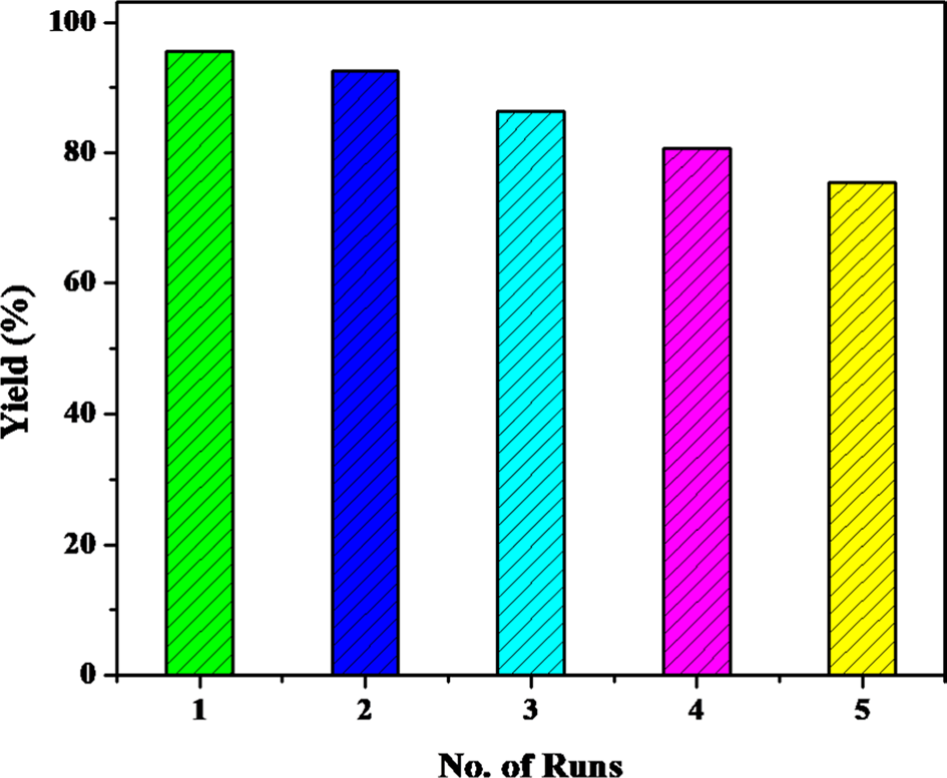
Recyclibility of NiCoO_2_ nanocomposite for the synthesis of Pyrazolopyrano[2,3-d]pyrimidine

The advantage of nanocomposites lies in their efficient reuse and recyclability, making them particularly valuable for sustainable material applications. After completion of the reaction, the reaction mixture was extracted with ethyl acetate. The organic layer was evaporated to obtain pyrazolopyrano[2,3-d]pyrimidine derivatives. The aqueous layer was filtered through Whatman filter paper No. 41 to separate the catalyst. Figure 16 shows that the catalyst could be reused for up to five consecutive cycles while retaining significant catalytic activity**.** A gradual decrease in product yield was observed with each successive cycle, which may be attributed to partial catalyst loss during recovery and handling, as well as possible surface deactivation of active catalytic sites (Figs. 17 and 18).

## Conclusion

In this study, a NiCoO_2_ nanocomposite was synthesized via a green route using *Moringa oleifera* leaf extract as a natural reducing and stabilizing agent. The nanocomposite exhibited high crystallinity and phase purity, with a crystallite size of 30 nm as determined by XRD analysis and an average particle size of about 54 nm as observed from SEM studies. Among the ten catalysts examined, NiCoO₂ demonstrated high catalytic performance, affording a 98% yield of pyrazolopyrano[2,3-d]pyrimidine derivatives within 20 min under ambient conditions in a water–ethanol (1:1) solvent system. The catalyst retained its activity over five consecutive cycles, indicating good stability and reusability. Molecular docking studies were carried out for all synthesized compounds against the 4COX enzyme, and the docking scores were compared with those of indomethacin as the reference compound. The docking results revealed that all compounds (1–11) exhibited higher binding scores than indomethacin. These docking results indicate favorable binding interactions with COX-2; however, experimental biological studies are required to validate their anti-inflammatory potential. Therefore, the synthesized compounds may serve as promising candidates for further biological investigations. Furthermore, both the nanocomposite and the synthesized heterocycles exhibited notable antimicrobial activity against the tested pathogenic microorganisms, highlighting their potential applications in sustainable catalysis and biomedical research.

## Supplementary Information

Supplementary material 1.

## Data Availability

All data generated during this study are included in this published article and its supplementary information files.
