# Supplementary material for: Bioinspired NiCoO2 nanocomposites derived from Moringa oleifera as a recyclable catalyst for the synthesis of pyrazolopyrano[2,3-d]pyrimidine with potent antimicrobial efficiency
Source: Discov Nano. 2026 Aug 3;21(1):372. doi: 10.1186/s11671-026-04800-6 (PMC13433697; doi:10.1186/s11671-026-04800-6)
Supplement: Supplementary file 1 [file 11671_2026_4800_MOESM1_ESM.pdf]

Bioinspired $NiCoO_2$ nanocomposites derived from *Moringa oleifera* as a recyclable catalyst for the synthesis of Pyrazolopyrano[2,3-d]pyrimidine with potent antimicrobial efficiency

Prakash Kokare[a,b], Avdhut Kadam[a], Ashwini More[a], Vijay Shinde[a], Vishvanath Ghanwat[a], Nilesh Pandit[a], Chaitali Bagade[b], Avinash Survase[c], Komal Mali[b], Santosh Kamble[d*,e]

[a] Department of Chemistry, Yashavantrao Chavan Institute of Science, Constituent college of Karmaveer Bhaurao Patil University, Satara-415001, Maharashtra, India

[b] Department of Chemistry, Shivaji University, Kolhapur, 416004, Maharashtra, India

[c] Department of Microbiology, Yashavantrao Chavan Institute of Science, Constituent college of Karmaveer Bhaurao Patil University, Satara-415001, Maharashtra, India

[d*] Department of Chemistry, Sadguru Gadage Maharaj College, Karad,415124, Maharashtra, India

[e] Karmaveer Bhaurao Patil University, Satara-415001, Maharashtra, India

[*]Corresponding Author: Santosh Kamble: santosh.san143@gmail.com

Orcid Id: 0000-0002-4668-1628

Sr.No.	Content	Page
1.	General	2
2.	Figure1.FTIR spectrum of 3-methyl-4-phenyl-4,8-dihydropyrazolo[4’,3’:5,6] pyrano[2,3-d]pyrimidine-5,7(1H,6H)-dione [TableNo.3,Entry-2]	3
3.	Figure 2.^{1}H NMR of 3-methyl-4-phenyl-4,8-dihydropyrazolo[4’,3’:5,6] pyrano[2,3-d]pyrimidine-5,7(1H,6H)-dione [TableNo.3,Entry-2]	4
4.	Figure 3.^{13}C NMR spectrum of 3-methyl-4-phenyl-4,8-dihydropyrazolo[4’,3’:5,6] pyrano[2,3-d]pyrimidine-5,7(1H,6H)-dione [TableNo.3,Entry-2]	5
5.	Figure 4.Mass spectrum of 3-methyl-4-phenyl-4,8-dihydropyrazolo[4’,3’:5,6] pyrano[2,3-d]pyrimidine-5,7(1H,6H)-dione [Table No.3,Entry-2]	6
6.	Figure 5.FTIR spectrum of 3-Methyl-4-(4-nitrophenyl)-4,8-dihydropyrazolo [3′,4′:5,6]Pyrano[2,3-d]pyrimidine5,7(1H,6H)-dione [TableNo.3,Entry-3]	7
7.	Figure 6.^{1}H NMR of 3-Methyl-4-(4-nitrophenyl)-4,8-dihydropyrazolo[3′,4′:5,6] Pyrano[2,3-d]pyrimidine5,7(1H,6H)-dione [TableNo.3,Entry-3]	8
8.	Figure 7.^{13}C NMR spectrum of 3-Methyl-4-(4-nitrophenyl)-4,8-dihydropyrazolo [3′,4′:5,6]Pyrano[2,3-d]pyrimidine5,7(1H,6H)-dione [TableNo.3,Entry-3]	9
9.	Figure 8.Mass spectrum of 3-Methyl-4-(4-nitrophenyl)-4,8-dihydropyrazolo [3′,4′:5,6]Pyrano[2,3-d]pyrimidine5,7(1H,6H)-dione [Table No. 3,Entry-3]	10
10.	Figure 9.FTIR spectrum of 4-(4-chlorophenyl)-3methyl-4,8-dihydropyrazolo [3′,4′:5,6]Pyrano[2,3-d]pyrimidine5,7(1H,6H)-dione [TableNo.3,Entry-4]	11
11.	Figure 10.^{1}H NMR of 4-(4-chlorophenyl)-3methyl-4,8-dihydropyrazolo[3′,4′: 5,6]Pyrano[2,3-d]pyrimidine5,7(1H,6H)-dione [TableNo.3,Entry-4]	12
12.	Figure 11.^{13}C NMR spectrum of 4-(4-chlorophenyl)-3methyl-4,8-dihydropyrazolo [3′,4′:5,6]Pyrano[2,3-d]pyrimidine5,7(1H,6H)-dione [TableNo.3,Entry-4]	13
13.	Figure 12.Mass spectrum of 4-(4-chlorophenyl)-3methyl-4,8-dihydropyrazolo [3′,4′:5,6]Pyrano[2,3-d]pyrimidine5,7(1H,6H)-dione [Table No. 3,Entry-4]	14

General:

Synthesisofpyrazolopyrano[2,3-d]pyrimidine:

In a 50 ml round-bottom flask, equimolar amounts of aromatic aldehyde (1mmol), ethylacetoacetate (1mmol), hydrazine hydrate (1mmol) and barbuteric acid (1mmol) were combined with $NiCoO_2$ nanocomposite (5 mol%) and dissolved this mixture in 10 ml of solvent system (1:1 water: ethanol), and the reaction was carried out under ultrasonication. The progress of reaction was monitored by TLC. After completion of reaction, the ensuing product was subjected to structural analysis using FT-IR, ^{1}H NMR, and ^{13}C NMR spectroscopy.

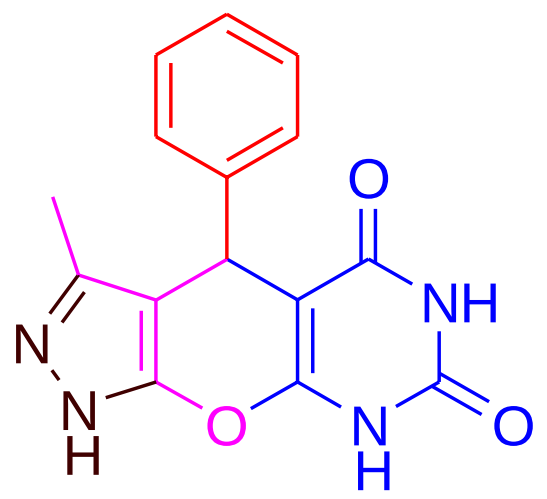

[TableNo.3, Entry-2]3-methyl-4-phenyl-4,8-dihydropyrazolo[4',3':5,6]pyrano[2,3-d]pyrimidine-5,7(1H,6H)-dione

FTIR (KBr) – 600cm^{-1}, 1100 cm^{-1}, 1400 cm^{-1}, 1590 cm^{-1}, 2250 cm^{-1}, 2900 cm^{-1}, 3400cm^{-1}.

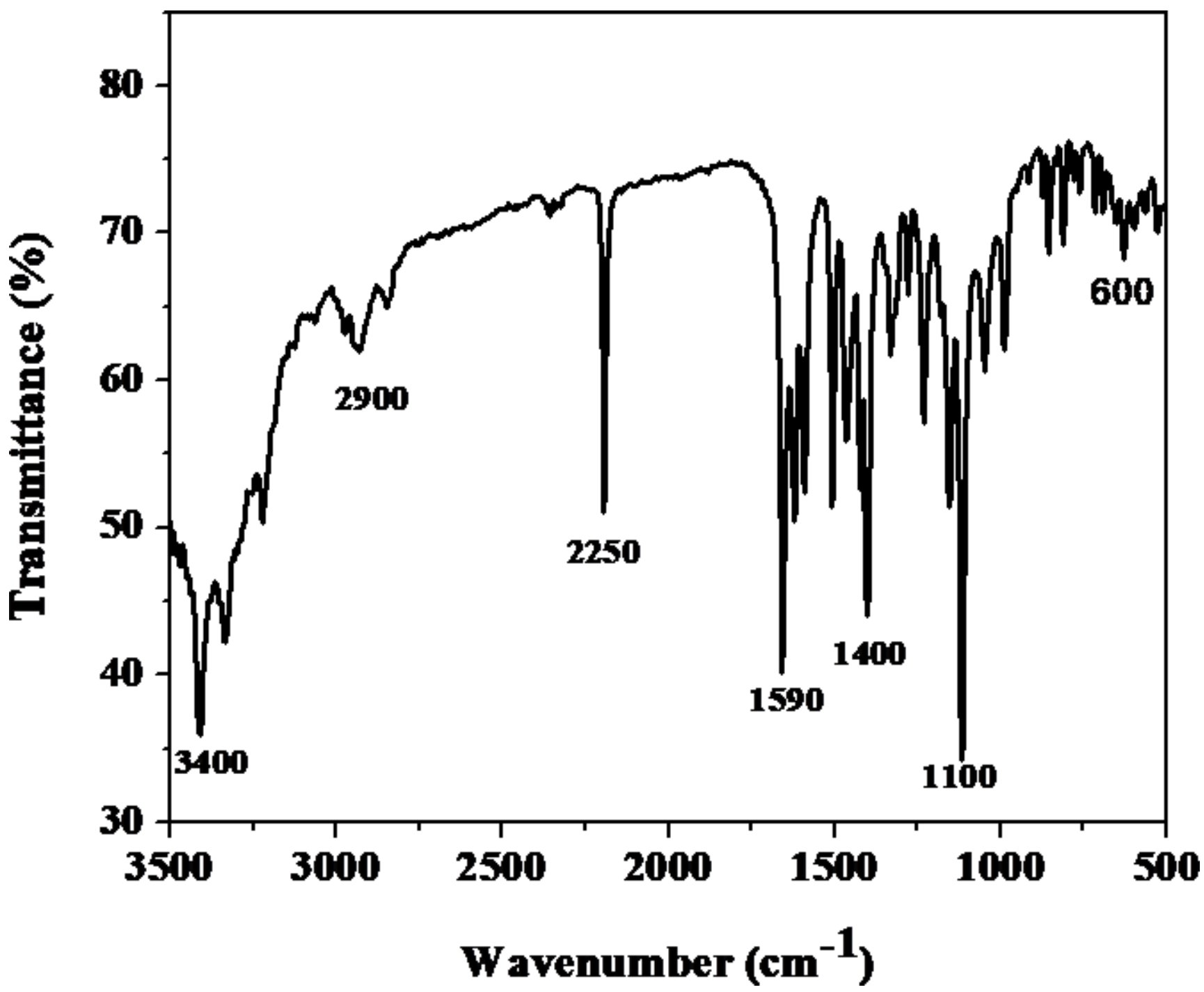

Figure1.FTIR spectrum of 3-methyl-4-phenyl-4,8-dihydropyrazolo[4',3':5,6]pyrano[2,3-d]pyrimidine-5,7(1H,6H)-dione

^{1}H NMR(400 MHz, DMSO) δ11.21 (s, 1H, NH), 10.12(s, 2H, NH), 7.18 (t, J=6.8 Hz, 2H,Ar-H), 7.13(d, *J*=6.8 Hz,2H, Ar-H), 6.90(t, J=6.8Hz 1H, Ar-H), 5.41 (s,1H, CH), 2.18(s,3H, CH_3).

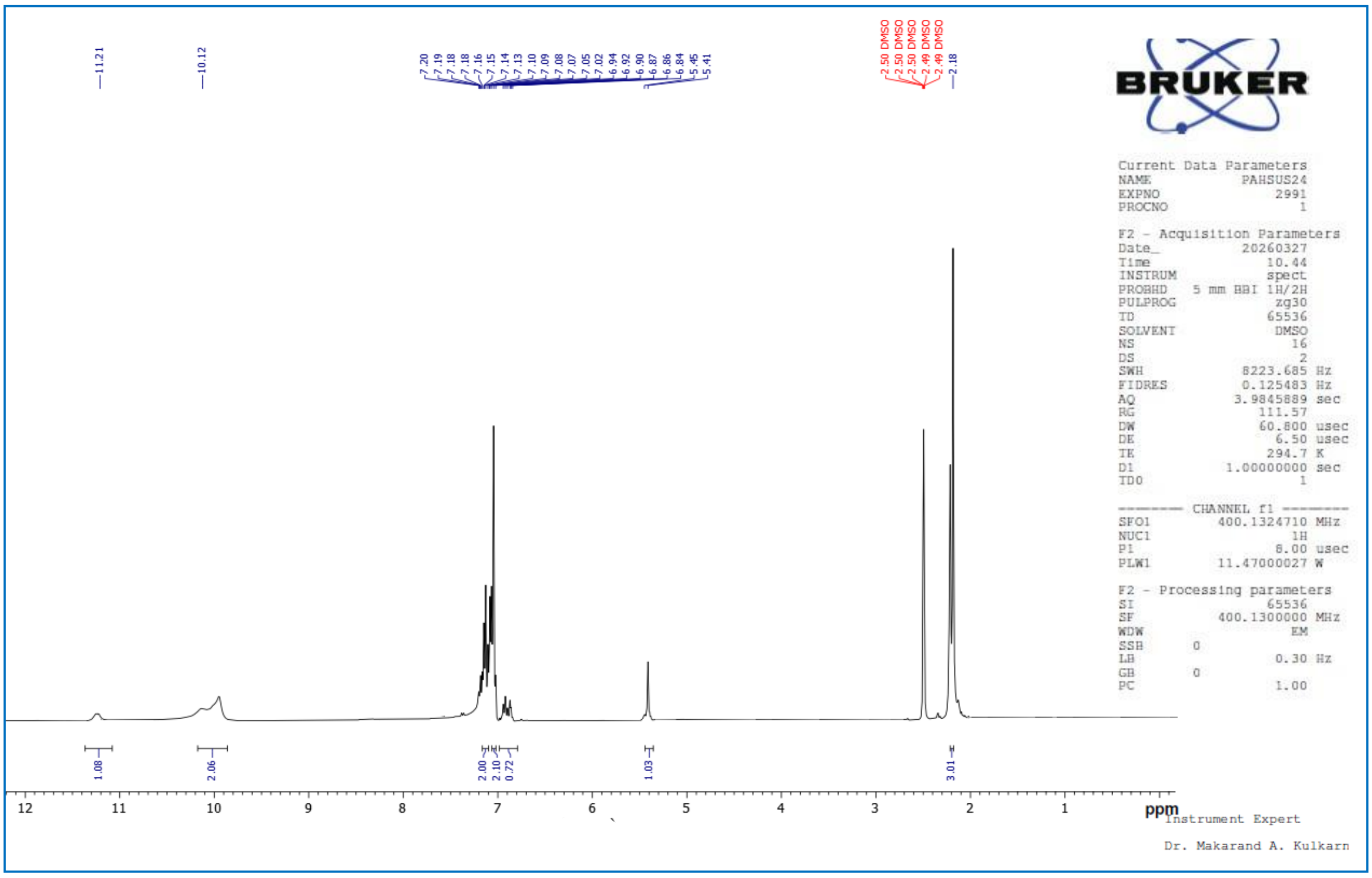

Figure2.^{1}H NMR of 3-methyl-4-phenyl-4,8-dihydropyrazolo[4',3':5,6]pyrano[2,3d]pyrimidine-5,7(1H,6H)-dione

^{13}C NMR (101 MHz, DMSO) δ170.09, 161.07, 152.98, 151.15,145.57, 142.33, 128.30, 127.08,125.46, 104.99, 92.62, 32.57, 10.61.

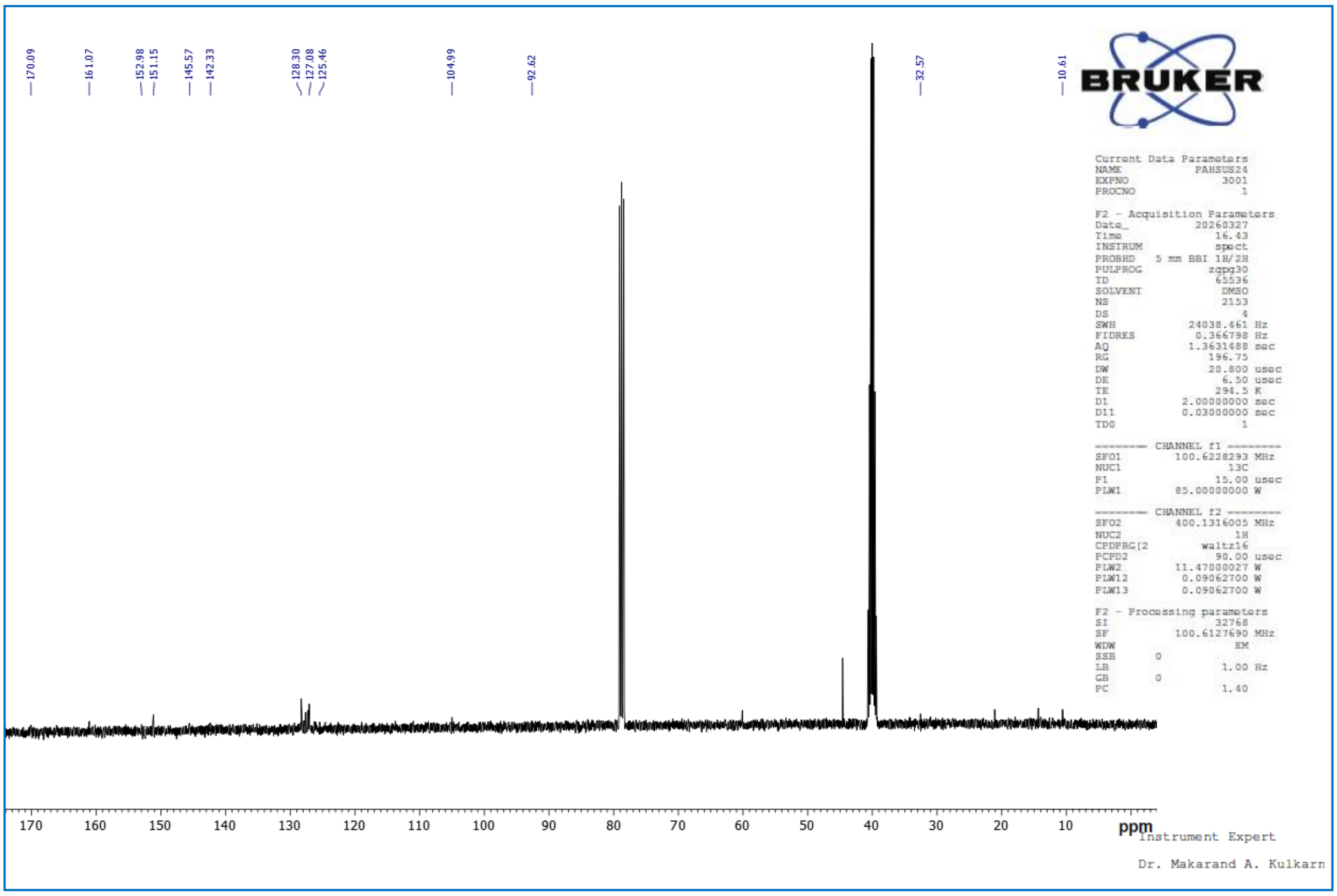

Figure3.^{13}C NMR spectrum of 3-methyl-4-phenyl-4,8-dihydropyrazolo[4’,3’:5,6]pyrano[2,3-d]pyrimidine-5,7(1H,6H)-dione

The mass spectrum of 3-methyl-4-phenyl-4,8-dihydropyrazolo[4',3':5,6]pyrano[2,3-d]pyrimidine-5,7(1H,6H)-dione shows a molecular ion peak at m/z ≈ 360**,** confirming the expected molecular weight of the compound. The presence of a single dominant peak with negligible fragmentation indicates a highly stable fused heterocyclic framework with resistance to breakdown under ionization conditions.

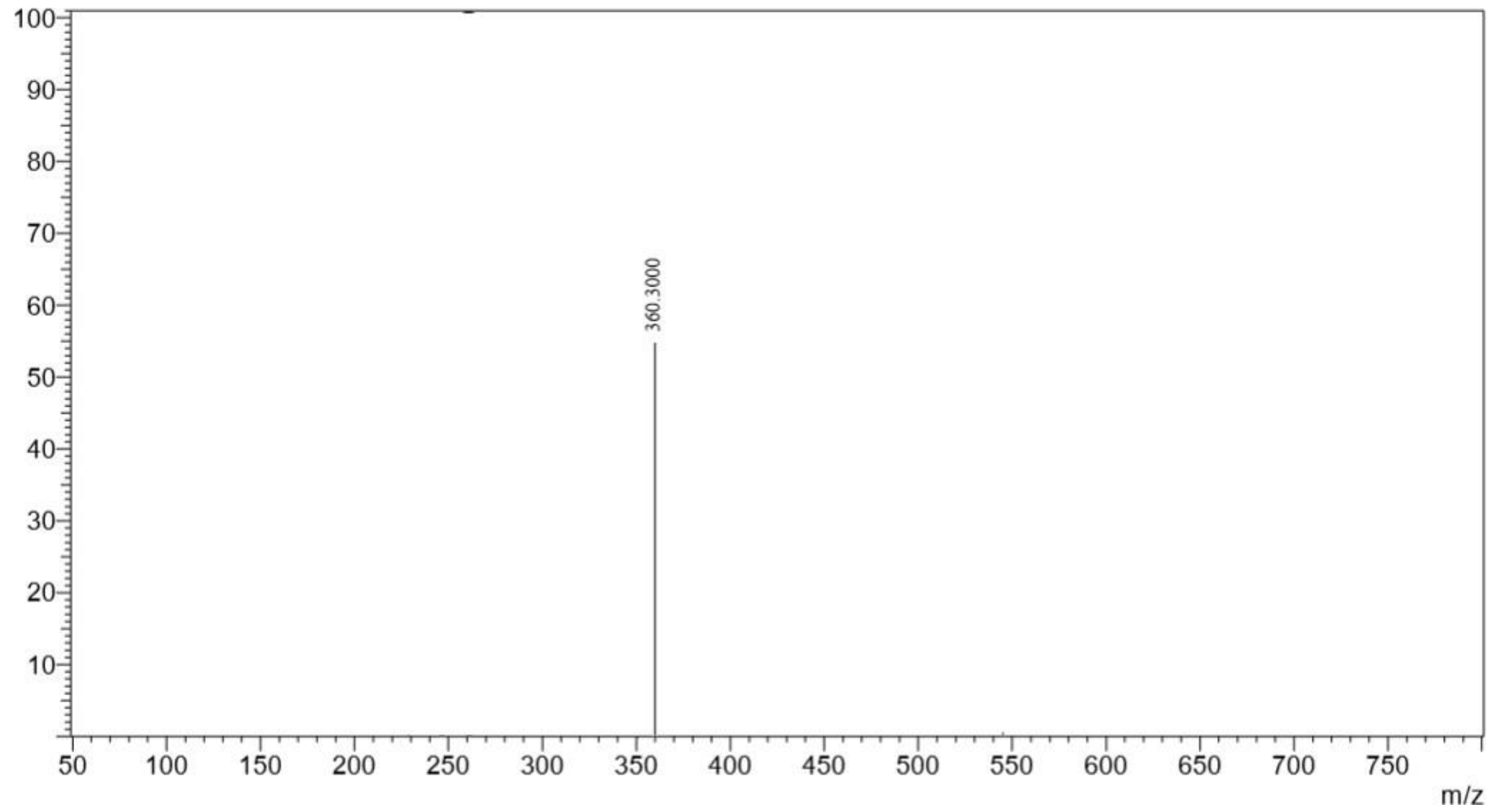

Figure4.Mass Spectrum of 3-methyl-4-phenyl-4,8dihydropyrazolo[4',3':5,6]pyrano[2,3-d]pyrimidine-5,7(1H,6H)-dione

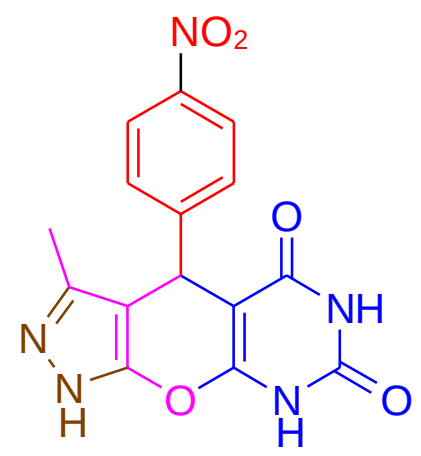

[TableNo.3,Entry-3]3-Methyl-4-(4-nitrophenyl)-4,8-dihydropyrazolo[3′,4′:5,6]Pyrano[2,3-d] pyrimidine 5,7(1H,6H)-dione

FTIR (KBr):600cm^{-1}, 1100cm^{-1}, 1400cm^{-1}, 1600cm^{-1}, 2900 cm^{-1}, 3400cm^{-1}.

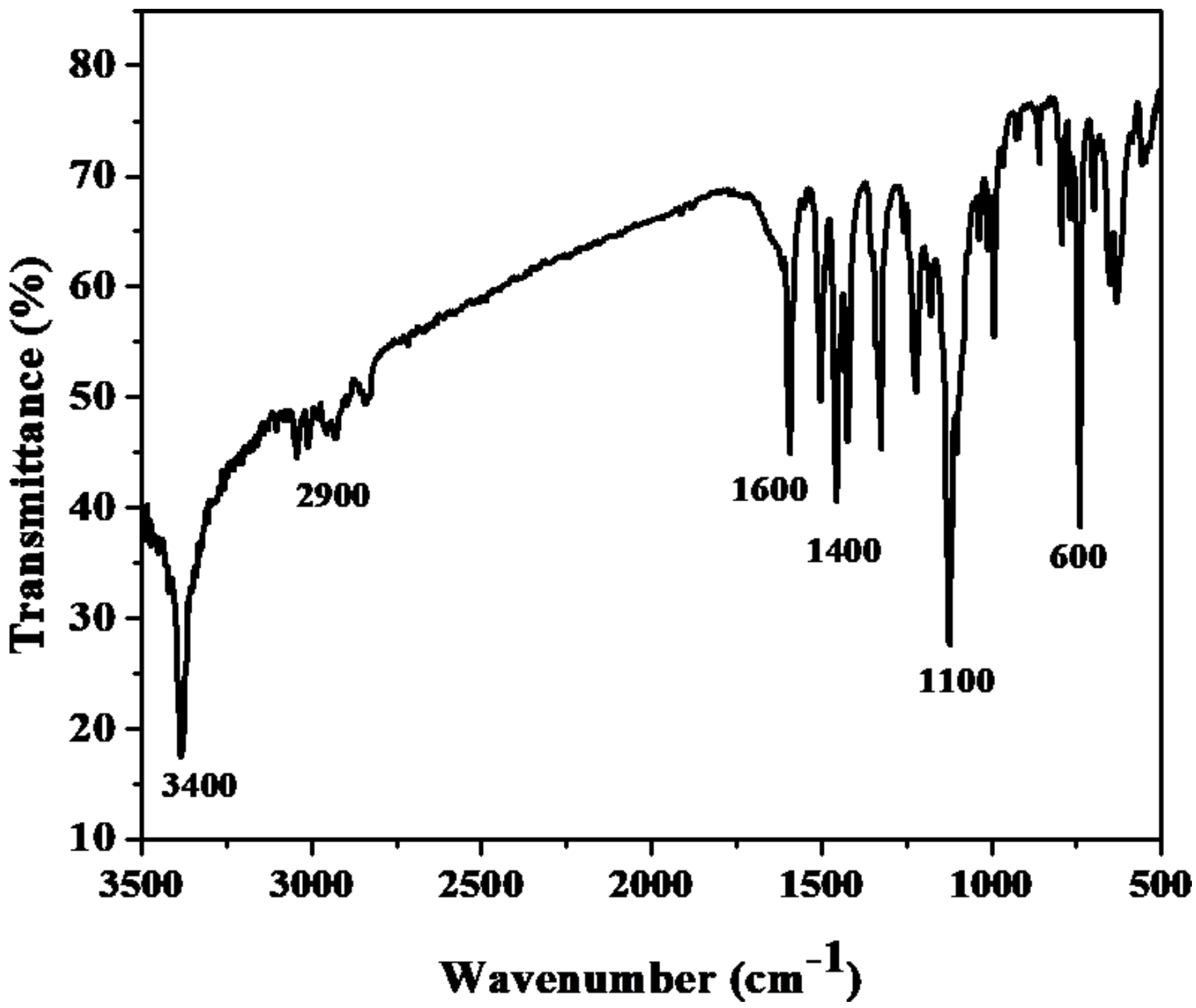

Figure5. FTIR spectrum of 3-Methyl-4-(4-nitrophenyl)-4,8-dihydropyrazolo[3′,4′:5,6]Pyrano [2,3-d] pyrimidine 5,7(1H,6H)-dione

^{1}H NMR (400 MHz, DMSO) δ11.16 (s,1H,NH), 10.11 (s,2H, NH),8.04 (d,J=8Hz, 2H, Ar-H),7.34 (d, J=7.6Hz, 2H, Ar-H), 5.52(s, 1H, CH), 2.23(s, 3H, CH_3).

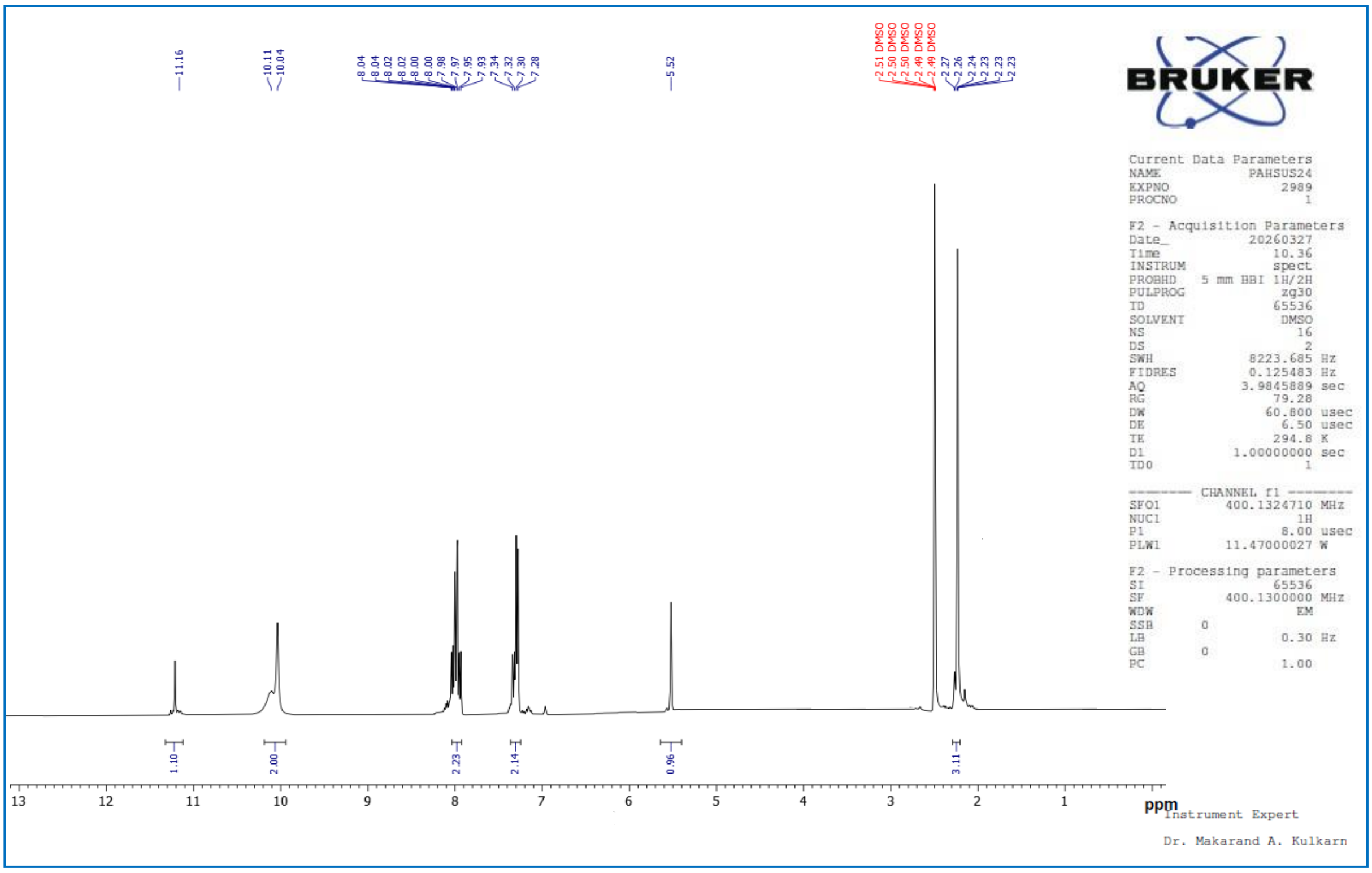

Figure6.^{1}H NMR of 3-Methyl-4-(4-nitrophenyl)-4,8-dihydropyrazolo[3′,4′:5,6]Pyrano[2,3-d] pyrimidine 5,7(1H,6H)-dione

^{13}C NMR (101 MHz, DMSO)δ165.40, 160.33, 155.11, 151.55,151.16, 145.90, 143.67, 128.23, 123.12, 104.11, 91.12, 31.60, 10.43.

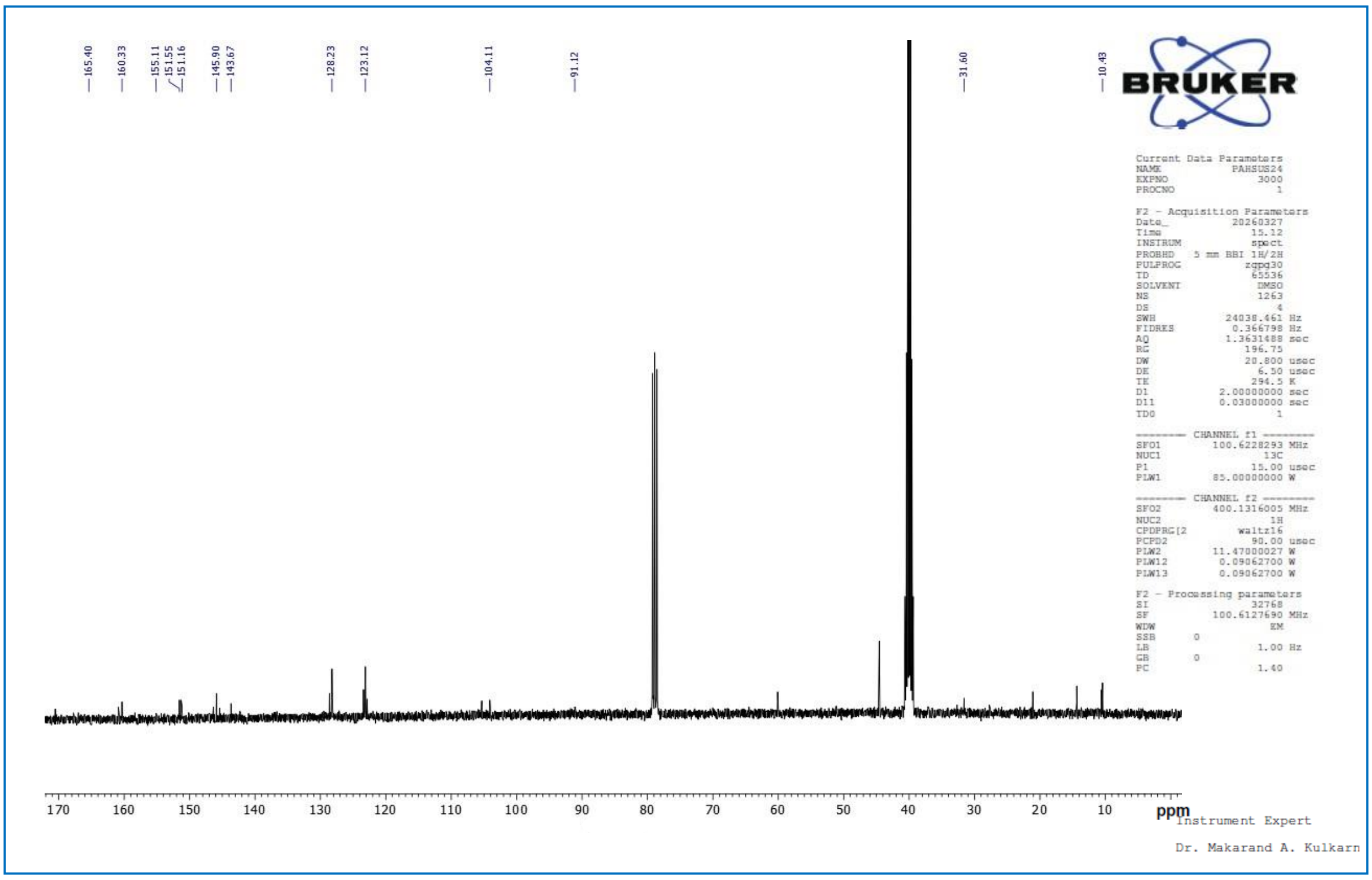

Figure7.^{13}C NMR of 3-Methyl-4-(4-nitrophenyl)-4,8-dihydropyrazolo[3′,4′:5,6]Pyrano [2,3-d] pyrimidine 5,7(1H,6H)-dione

The mass spectrum of 3-methyl-4-(4-nitrophenyl)-4,8-dihydropyrazolo[3′,4′:5,6]pyrano[2,3-d]pyrimidine-5,7(1H,6H)-dione shows a prominent molecular ion peak at m/z ≈ 341, confirming the molecular weight of the compound. The presence of a single intense peak with minimal fragmentation indicates a highly stable fused heterocyclic structure, with little breakdown under ionization conditions.

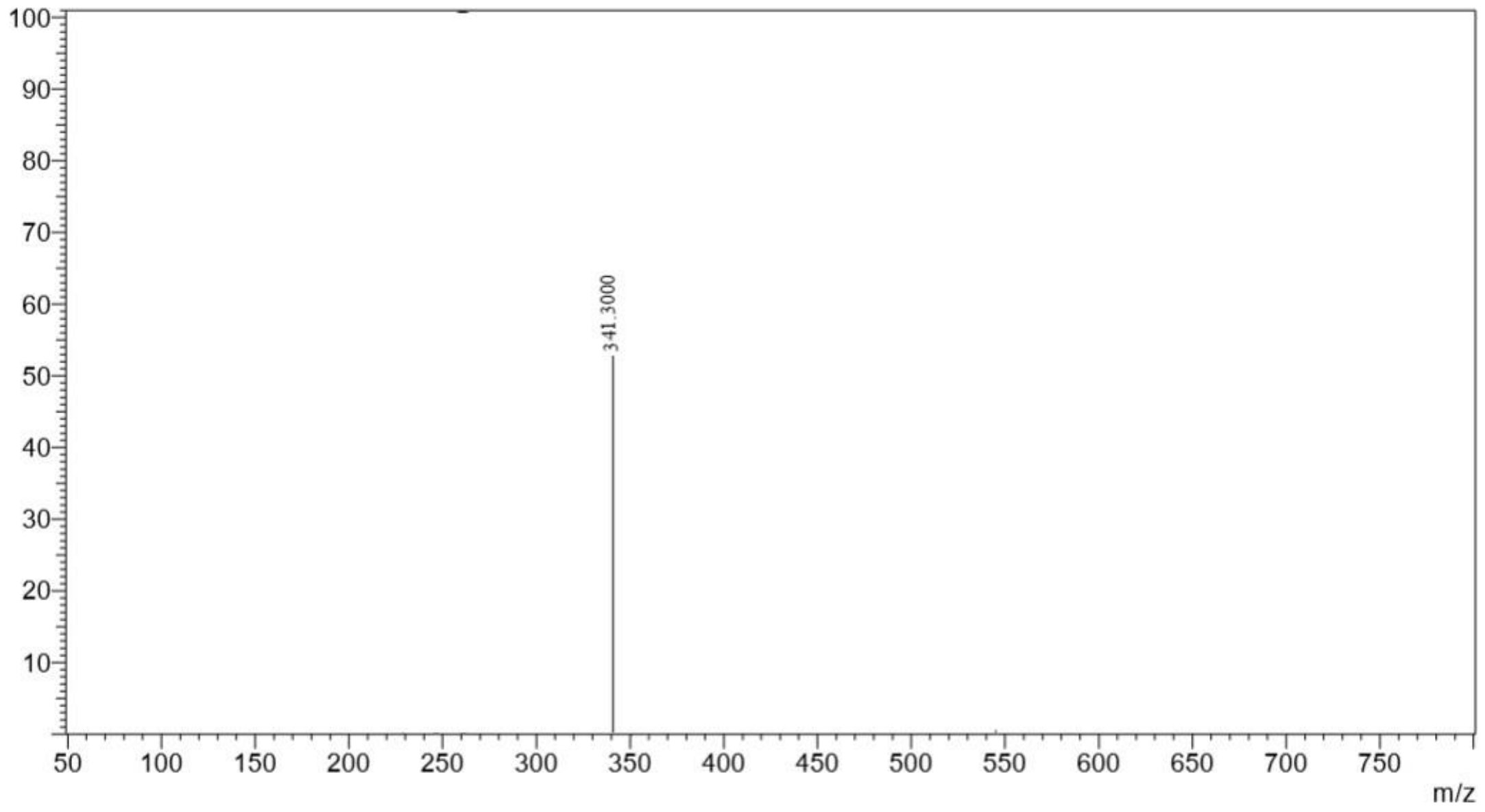

Figure8.Mass spectrum of 3-Methyl-4-(4-nitrophenyl)-4,8-dihydropyrazolo[3′,4′:5,6] Pyrano [2,3-d] pyrimidine 5,7(1H,6H)-dione

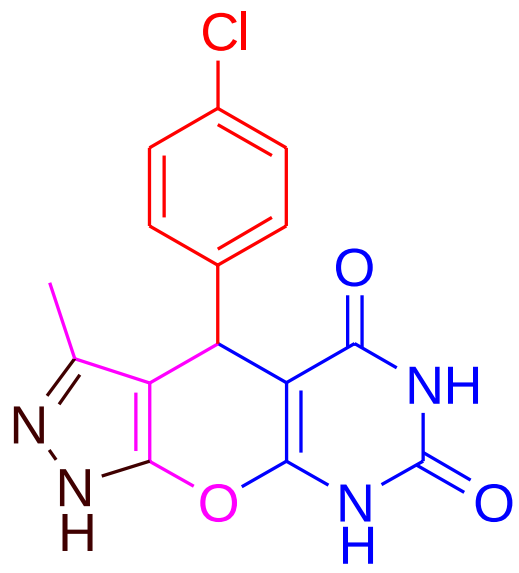

[TableNo.3,Entry-5]4-(4-chlorophenyl)-3methyl-4,8-dihydropyrazolo[3′,4′:5,6]Pyrano[2,3-d]pyrimidine5,7(1H,6H)-dione

FTIR (KBr):600cm^{-1}, 1100cm^{-1}, 1400 cm^{-1}, 1600cm^{-1}, 2100cm^{-1}, 2900cm^{-1}, 3400cm^{-1}.

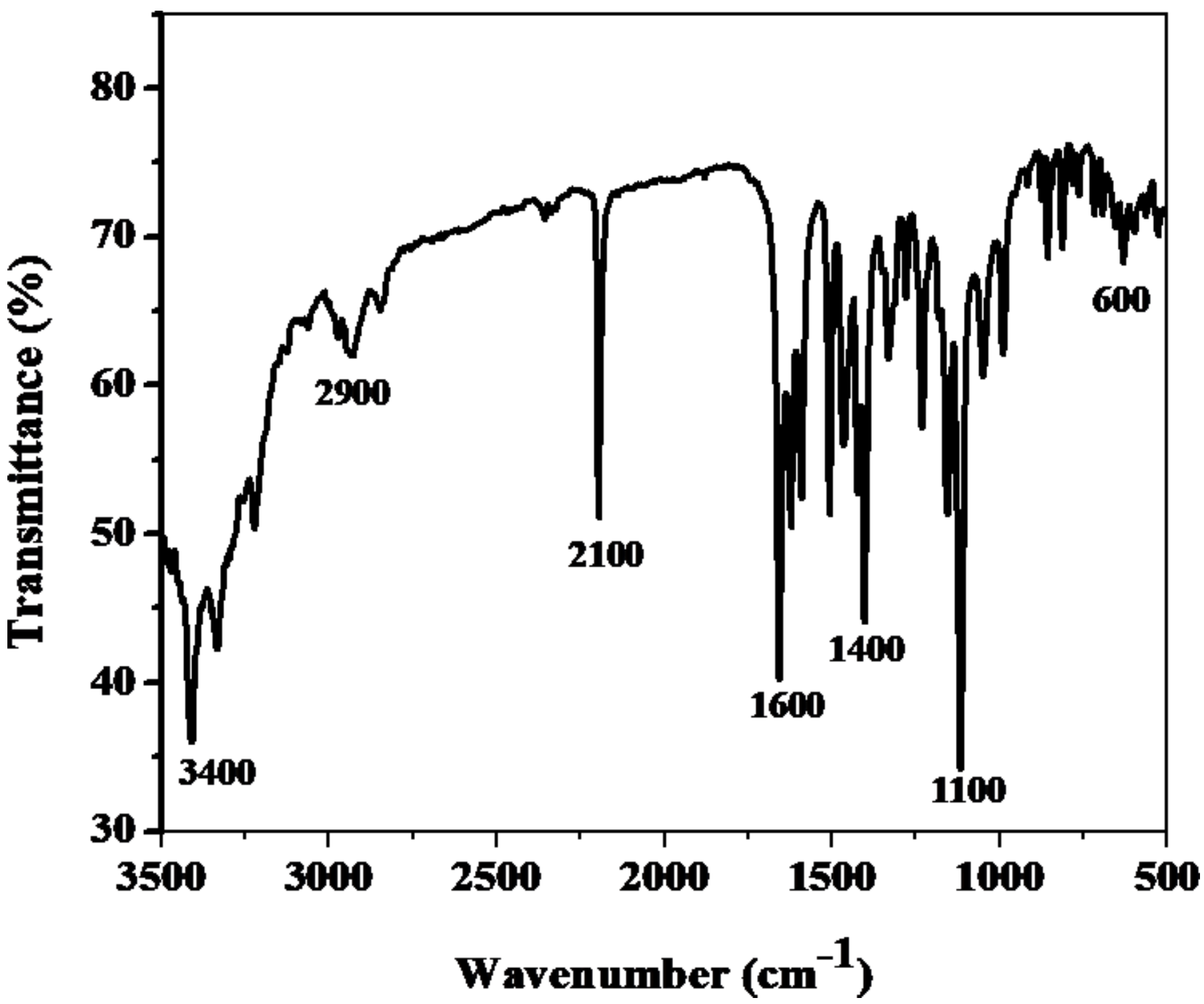

Figure9.FTIR spectrum of 4-(4-chlorophenyl)-3methyl-4,8-dihydropyrazolo[3′,4′:5,6] Pyrano [2,3-d] pyrimidine 5,7(1H,6H)-dione

[1]**H NMR (400 MHz, DMSO)δ**12.65 (s, 1H,NH),10.12 (s, 2H,NH), 7.91 (d,J=8 Hz, 2H, Ar-H), 7.05(d, J=7.2 Hz, 2H, Ar-H), 5.41(s, 1H,CH), 2.23 (d, *J*=12.2 Hz, 3H, CH_3).

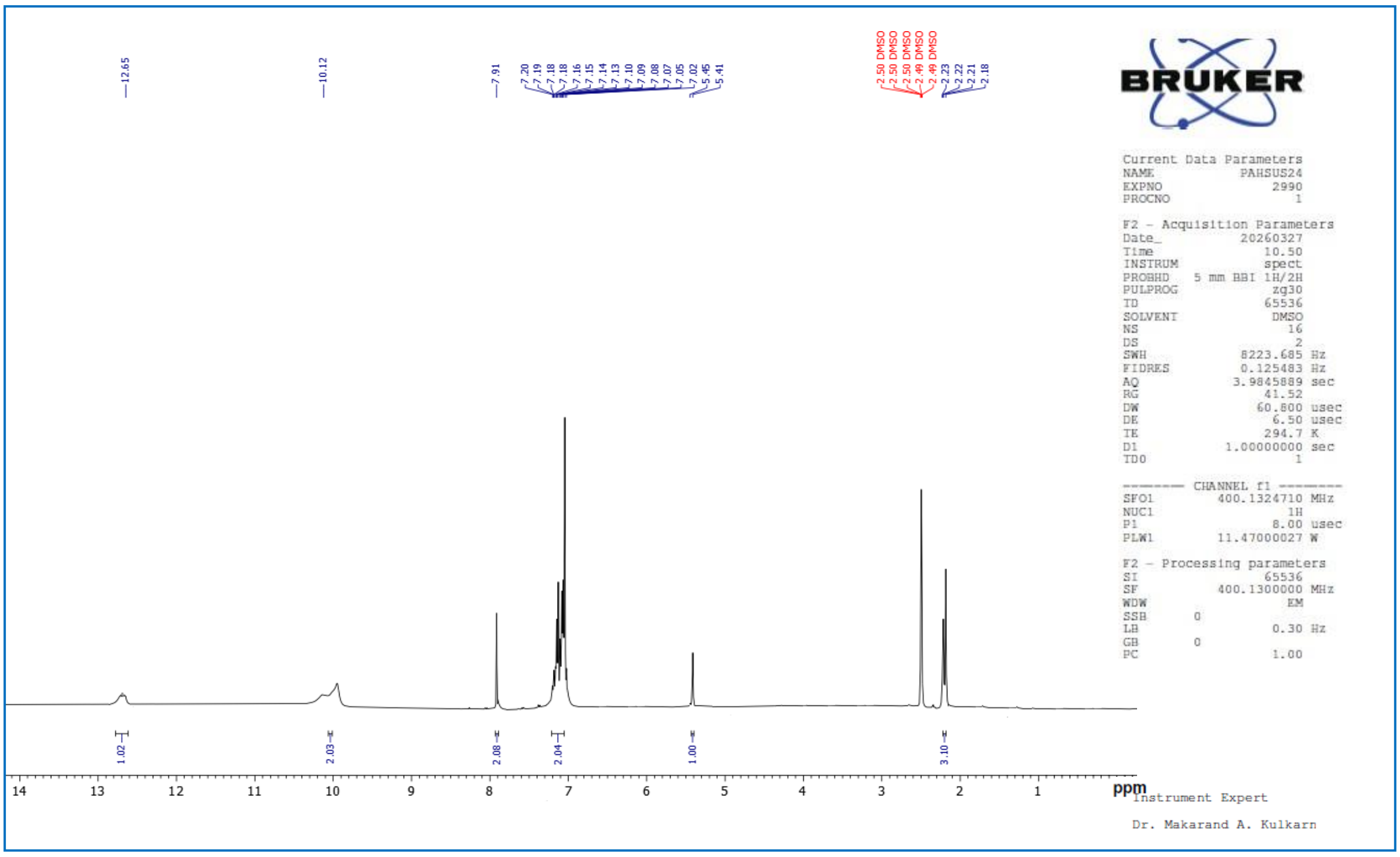

Figure10.[1]**H NMR of 4-(4-chlorophenyl)-3methyl-4,8-dihydropyrazolo[3′,4′:5,6]Pyrano [2,3-d] pyrimidine 5,7(1H,6H)-dione**

^{13}C NMR (101 MHz, DMSO) δ165.87, 160.99, 154.27, 151.69,151.20, 143.10, 140.97, 128.16, 127.53, 104.62, 91.48, 32.09, 14.35.

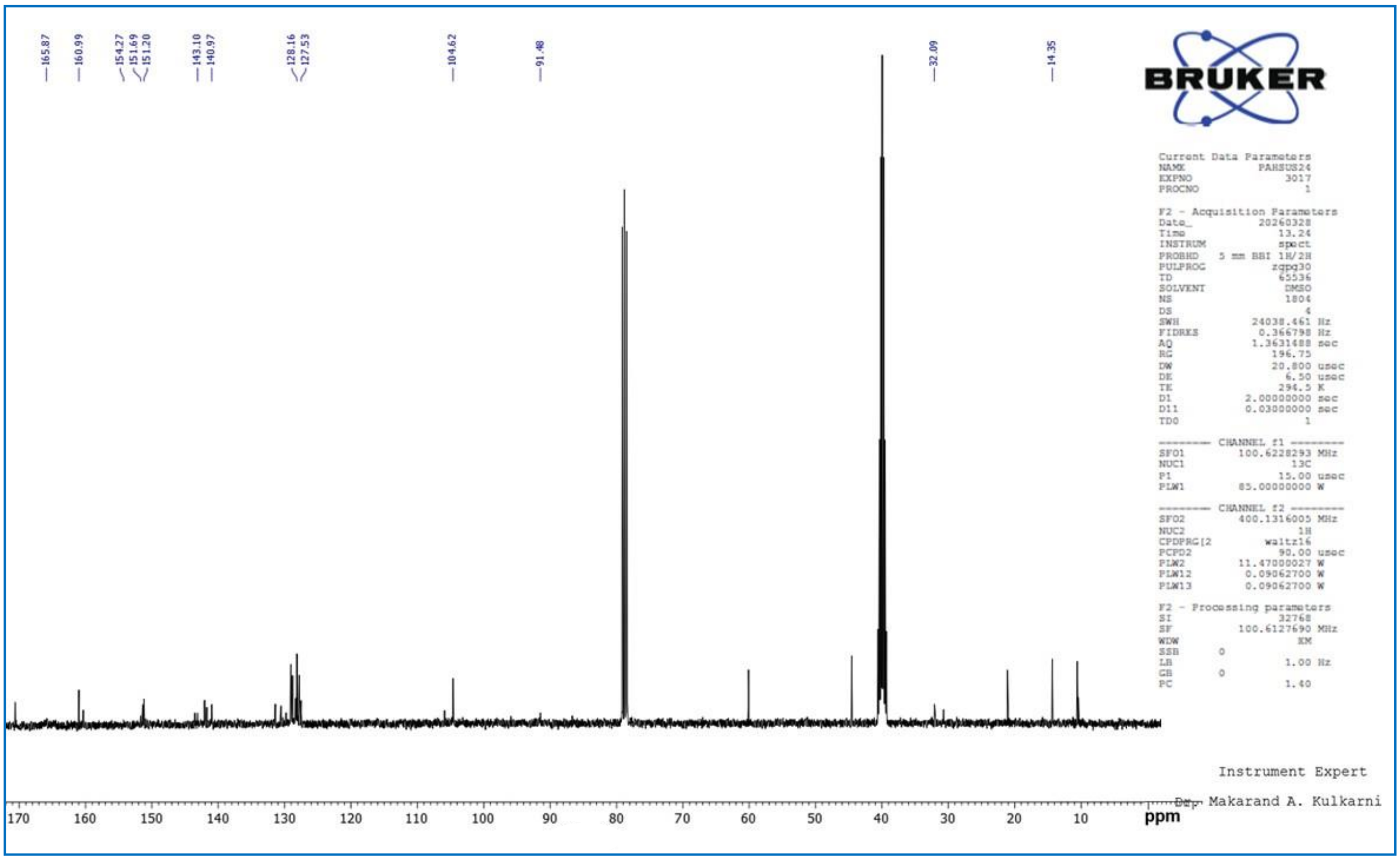

Figure11.^{13}C NMR of 4-(4-chlorophenyl)-3methyl-4,8-dihydropyrazolo[3′,4′:5,6]Pyrano[2,3-d] pyrimidine 5,7(1H,6H)-dione

The mass spectrum of 4-(4-chlorophenyl)-3-methyl-4,8-dihydropyrazolo[3′,4′:5,6]pyrano[2,3-d]pyrimidine-5,7(1H,6H)-dione shows a molecular ion peak at m/z ≈ 354, confirming the expected molecular weight of the compound. A prominent fragment peak at m/z ≈ 331 corresponds to the loss of a small neutral fragment (such as HCl or NH_3), while the limited fragmentation pattern indicates the high stability of the fused heterocyclic system.

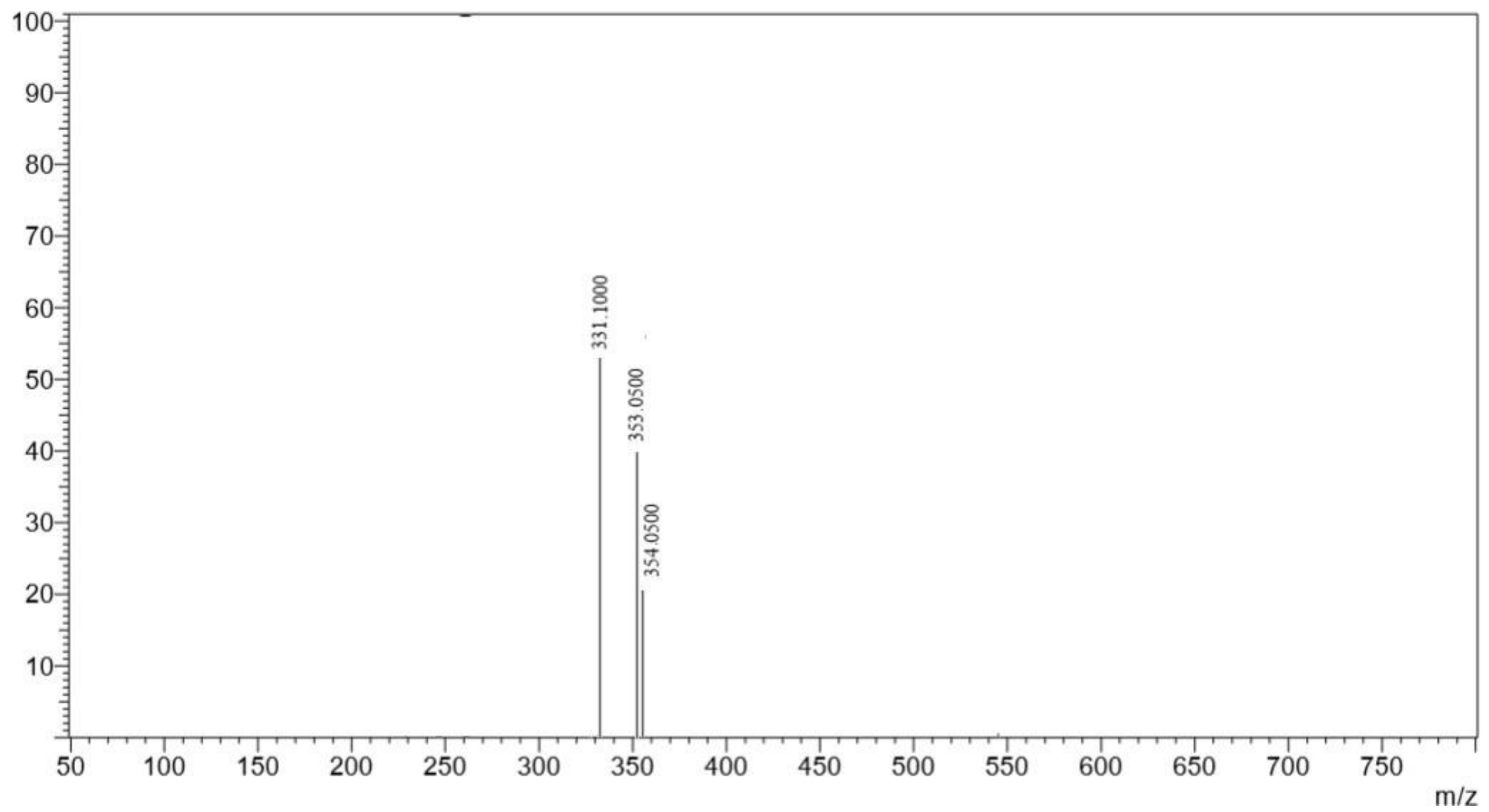

Figure12.Massspectrumof4-(4-chlorophenyl)-3methyl-4,8-dihydropyrazolo[3′,4′:5,6] Pyrano [2,3-d] pyrimidine 5,7(1H,6H)-dione
